# Supplementary material for: DNA metabarcoding reveals metacommunity dynamics in a threatened boreal wetland wilderness
Source: Proc Natl Acad Sci U S A. 2020 Mar 26;117(15):8539–45. doi: 10.1073/pnas.1918741117 (PMC7165428; doi:10.1073/pnas.1918741117)
Supplement: Supplementary File [file pnas.1918741117.sapp.pdf]

Supplementary Information Appendix 1: Multi-species occupancy modelling of freshwater macroinvertebrates in the Peace-Athabasca Delta based on morphological identification and DNA metabarcoding.

Alex Bush

26th June 2019

Contents

Input Data **2**
- Sampling sites . . . 2
- Environmental parameters . . . 2
  - Temperature . . . 3
  - Flood frequency and duration . . . 4
- Assemblage Data . . . 6
  - Morphological Data . . . 6
  - DNA Metabarcoding Data . . . 6
  - Exploration of community data . . . 8

Hierarchical Model Framework **9**
- Occupancy Modelling . . . 9
  - JAGS Models . . . 11
  - Convergence, model fit and variable selection . . . 13
  - N-mixture Model . . . 14

Results **16**
- Metacommunity size (gamma diversity) . . . 16
- Site richness (alpha diversity) . . . 17
- Occupancy and detectability . . . 19
  - CABIN vs. metabarcoding at family-level . . . 20
  - Taxa unique to one approach . . . 20
  - Family-level vs genus-level . . . 22
- Environmental effects . . . 23
- Compositional Turnover (beta diversity) . . . 26

This supplementary file describes the inputs and model structure for the analysis of occupancy of macroinvertebrates based on composition derived from morphological identification and DNA metabarcoding. Details of the Canadian Aquatic Biomonitoring Network's wetland protocol are available from Environment and Climate Change Canada online.

---

www.pnas.org/cgi/doi/10.1073/pnas.1918741117

Input Data

Sampling sites

Table S1.1: Coordinates of sampling sites analysed in this study (also displayed in Figure 1 in the main text).

Site Name	Site No.	Latitude	Longitude
Otter Crk.	1	58.60273	-111.5261
Mamawi Bay	3	58.56475	-111.5108
Mamawi Crk. Pond	4	58.50773	-111.5180
Child's R.	11	58.63840	-111.5965
Rat Lk.	14	58.87465	-111.3248
Egg Lk	33	58.88236	-111.3992
Rocher R.	37	58.83234	-111.2807
Horseshoe Slough	38	58.86389	-111.5816

During the initial development phase of the monitoring program, between 2012 and 2014, sampling took place twice a year in June and August, and the majority of wetland sites were sampled three times each (i.e. triplicate benthic samples). However, after the initial period of funding ended, the cost of multiple field surveys per year, and a high degree of replication was not sustainable. As a result, field surveys have since been conducted in August, and sample processing has been maintained to characterize inter-annual variability, rather than within-site replicate similarity or seasonal variability.
DNA samples were analysed with BE in 2011 [1], BE + F230 [1,2] in 2012-2013, BR5 + F230 in 2014-2016 [3,4]. There is a 3 base pair difference between the E and ArR5 primers (ArR5 has an additional IGG at the 3' end), but both the BE and BR5 ampicons target the same COI region (E: 5'-GTRATIGCICCIGCIARIAC;ArR5: 5'- GTRATIGCICCIGCIARIACIGG).

Table S1.2: Number of invertebrate samples collected from the same eight sites in the Peace-Athabasca Delta in northern Alberta between 2011 and 2016, processed either using traditional morphological identification based on the standard guidelines of the Canadian Aquatic Biomonitoring Network (CABIN), or by using DNA metabarcoding.

Year	2011	2012	2012	2013	2013	2014	2014	2015	2016	Total
Month	Aug	Jun	Aug	Jun	Aug	Jun	Aug	Aug	Aug	
CABIN	8	24	24	24	8	8	8	12	10	126
DNA	24	24	23	24	20	0	8	7	8	138

Environmental parameters

The Peace-Athabasca is remote, making it challenging to access sites and collect data, and to leave and retrieve recording devices. The system is naturally hydrologically dynamic with a high variation in the frequency of flooding among sites, and between years. If spring snowmelt is combined with an ice-jam, water levels can rapidly increase by 2 meters or more, which is sufficient for floodwaters to connect adjacent wetlands. The potential impacts of high flow and ice are that recording devices are often damaged or lost, even at the permanent monitoring stations. Below we show where those gaps occurred in the environmental records and how we related available data to describe the variation in site condition.

Temperature

Air temperature is publicly available online from Environment Canada meteorological station at Fort Chipewyan (Gauge #71305, -111.12 W, 58.77 N). The record for water temperature at each of our study sites however was discontinuous, and there was evidence of a site level effect on their relationship to air temperature (F=7.079, p=0.0008). Annual changes in water temperature were therefore predicted separately for each site, using mean air temperature when it exceeded 0'C. We tested a range of lag periods and functions, and found water temperature could be predicted with a reasonable degree of accuracy ($r2$>0.8) using only one or two covariates: the mean temperature of the preceding 3 and 14 day period. These models were subsequently used to predict continuous water temperature profiles for each site, and estimate three potential covariates:
1. the period of time each site will have been free of ice at the time of sampling (i.e. time >0'C prior to June or August surveys).
2. the mean water temperature the month prior to sampling
3. the maximum water temperature of the year prior to sampling

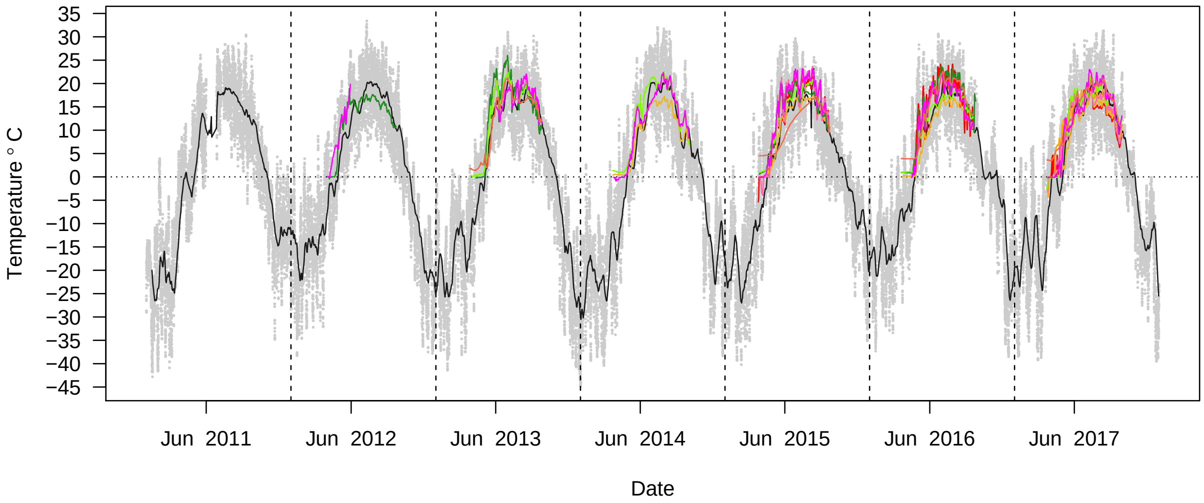

Figure S1.1: Range of air temperatures recorded hourly at Fort Chipewyan, adjacent to the PAD, between 2011 and 2017 (grey). This information was summarised to 3-day (not shown) and 14-day mean air temperatures (black line), and used to predict water temperature at each sampling site. The coloured lines indicate the periods for which we had records of water temperature at each site to fit those models.

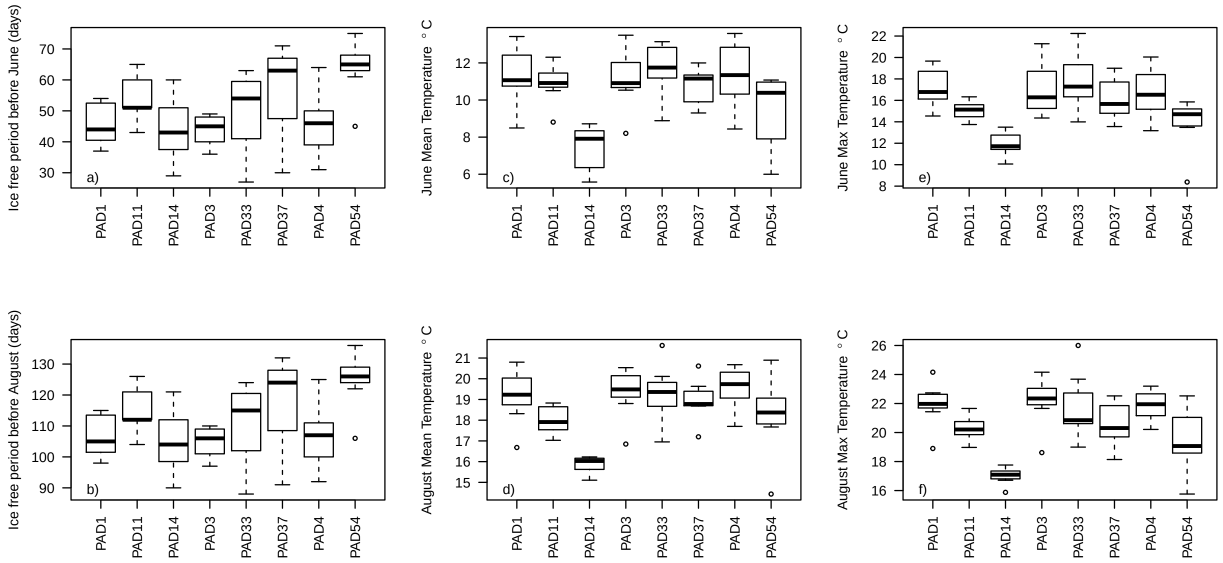

Figure S1.2: Site-level variation for the three covariates for water temperature regime used in our analysis divided between June and August: ice-free period (a and b), mean water temperature (c and d), and maximum water temperature (e and f).

Flood frequency and duration

Like temperature, our study made use of water level data collected by Environment and Climate Change Canada from the surveyed sites [5,6]. Environment and Climate Change Canada's HYDAT Water Survey Data Products are available at: https://www.canada.ca/en/environment-climate-change/services/water-overview/quantity/monitoring/survey/data-products-services.html). We could not calibrate those discontinuous site-specific hydrological profiles against a single continuously recording station because no such reference exists. There are currently 11 gauge stations operating on either the Peace and Athabasca rivers entering the PAD, in the PAD, and on the Slave river that drains from the delta. However, during the timeline of our study, it was rare for all gauges to be recording at the same time, and importantly, records of the flood peaks were missing from some locations. As a result, to predict water level at the specific wetlands that had been surveyed, we fit models to predict values for the gaps in the water level records of important gauging stations, using as inputs the values of other gauges that were still operating. A continuous estimate of water levels at each study site was subsequently based on all relevant gauging stations.

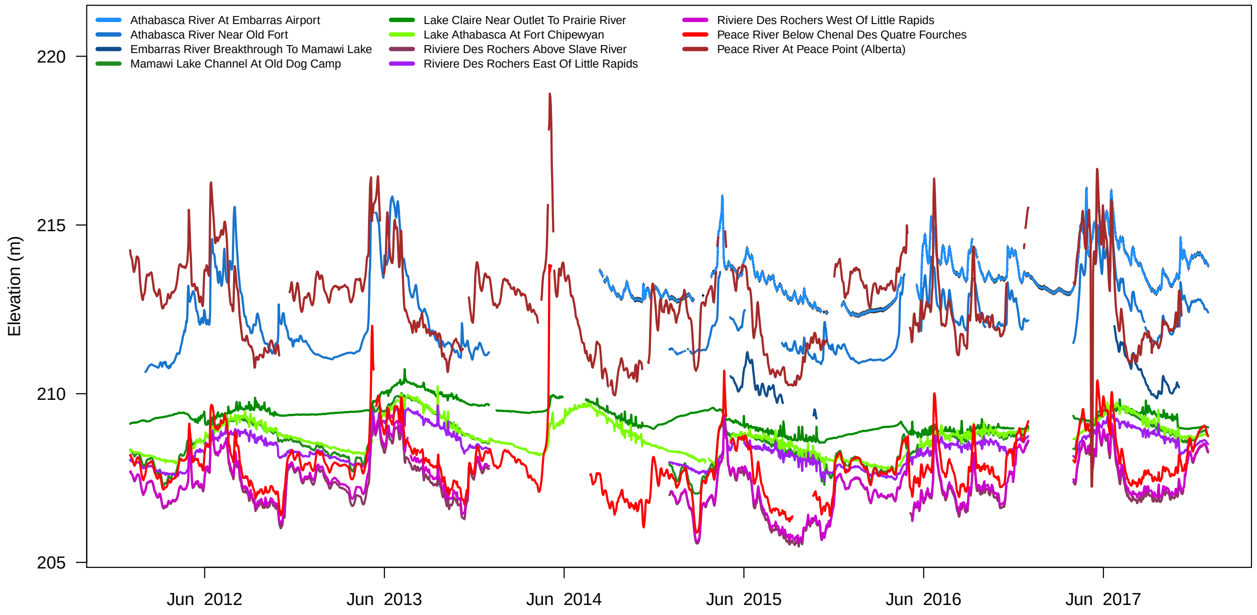

Figure S1.3: Water level at the 11 gauges associated with the Peace-Athabasca Delta between 2012 and 2017.

As was the case for the permanent flow gauges, there were gaps in the records available for water level in each of our study sites (Figure S1.4). It is worth noting that the delta is extensive because the landscape is very flat, and often small changes in water elevation are enough to connect main channels to adjacent wetlands. Furthermore, note that there were no measurements of water-level at these particular sites during the first season of field surveys in 2011. Although surveys in 2017 do not form part of the analysis in this study, we did take advantage of the water-level records from 2017 in order to improve our estimates of site-level hydrology.

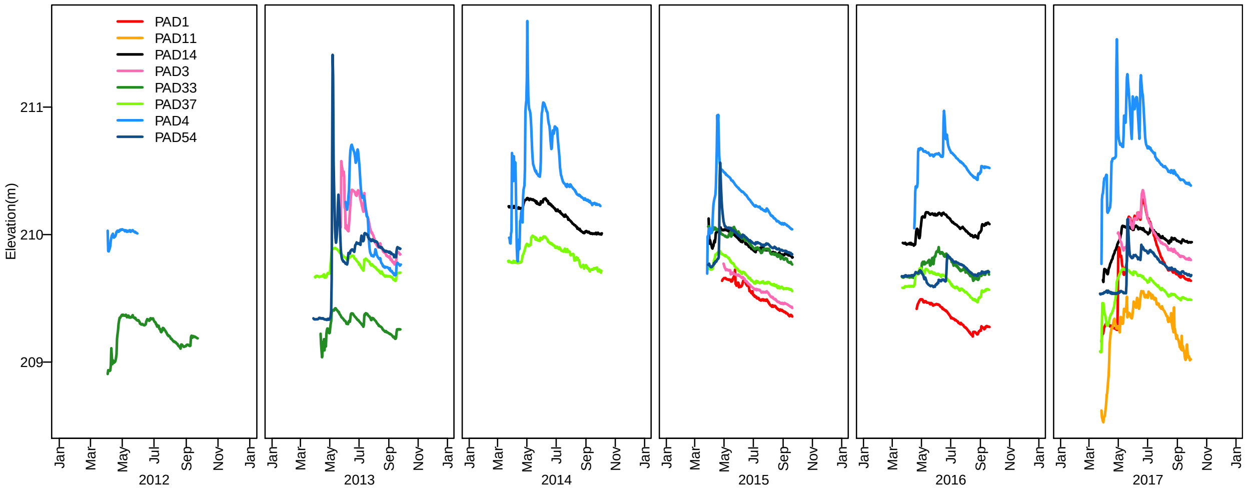

Figure S1.4: Record of water level from loggers deployed at the eight study sites between 2012 and 2017.

Data on the water-level at our study sites was most related to water level at gauging stations during floods, because this is when the water in the channels and adjacent wetlands was most likely connected. Thus our aim was to predict when and how often river levels exceed the wetland spill elevation, and for how long, whereas a model of the precise water level within our study site was beyond our needs. Models were calibrated to recover dynamics whose peak flows corresponded to rapid changes in water level within the wetland (Figure S1.3 and S4). We were therefore able to characterise inter-annual variation in the hydrology and connectivity of each wetland based on the frequency and duration of flooring in the spring and summer months before they were surveyed. Note the duration of time a site was flooded in a given year was positively associated

with the frequency of flooding (r=0.57).

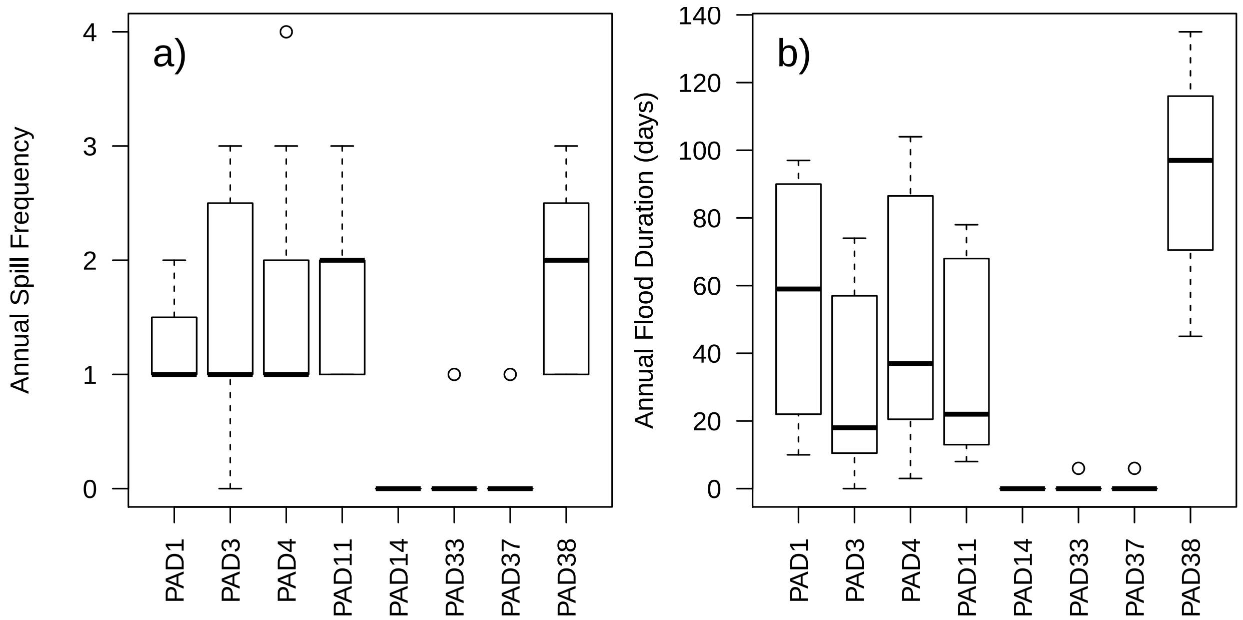

Figure S1.5: Variability in estimated a) annual flood frequency, and b) flood duration, per site in this study.

Assemblage Data

Morphological Data

Wetland invertebrate samples identified based on morphological characteristics followed standard protocols that mirror the traditional guidelines used to assess river and stream kick samples in the Canadian Aquatic Biomonitoring Network (CABIN), and hence will hereafter be referred to as the CABIN samples. The taxonomic resolution of CABIN data varies among invertebrate groups depending on the difficulty of identification, the perceived importance of a particular group and the expertise of the taxonomist. Thus although most of the 74 taxa identified were at the family-level, and subsequent analysis will refer to the CABIN data as being at family-level, there were some exceptions:
1. Information on the four sub-families of Chironomidae was retained; namely Chironominae, Orthocladiinae, Tanypodinae & Tanytarsini.
2. The Subclass Hirudinea was retained.
3. The Subclass Oligochaeta was retained.

DNA Metabarcoding Data

The sequence data is available from the NCBI Sequence Read Archive, BioProject ID: PRJNA603969.

Raw paired-end Illumina MiSeq reads were processed using the SCVUC v 2.1 COI metabarcode pipeline available from https://github.com/Hajibabaei-Lab/SCVUC_COI_metabarcode_pipeline
Jobs were run in parallel using GNU parallel [7]. Briefly, reads were paired using SEQPREP v1.2 ensuring a minimum Phred quality of 20 and a minimum overlap of 25 bp [8]. Primers were trimmed using CUTADAPT v1.15 ensuring a minimum trimmed sequence length of 150 bp, mimimum Phred quality of 20 at the ends, and no more than 3 ambiguous bases allowed [9]. Sequences were dereplicated using VSEARCH 2.7.0 '–derep_fulllength' [10]. Unique sequences were denoised using USEARCH v10.0.240 with the unoise3 algorithm

[11]. Denoising involves the removal of rare sequence clusters, potential PhiX carryover, sequences with putative errors, and putative chimeric sequences. We defined rare sequence clusters as containing only one or two sequences. An ESV x sample table was build using VSEARCH '–usearch_global' setting the identity to 1.0 (100% similarity). Taxonomic assignments were performed using the COI Classifier v3 available from https://github.com/terrimporter/CO1Classifier [12]. This classifier uses the Ribosomal Database Project naive Bayesian classifier with a COI reference set mined from GenBank [13]. We used the recommended cutoffs to ensure taxonomic assignments are 99% correct, assuming the query taxa are represented in the database.

As the sampling approach entails disturbance of emergent vegetation, it is common for wetland samples to contain a variety of invertebrates with a terrestrial, rather than freshwater origin. For the purposes of this study we focused solely on freshwater taxa, and taxa whose lifecycle was not known to include an aquatic freshwater phase were discarded. The resulting dataset included 263 invertebrate genera among 109 families, and consistent with most ecological studies, many taxa were rarely observed, and few were common. Those families and genera we could confidently identify were among the 29,099 and 15,587 unique sequences returned by the BR5 and F230 respectively. As Figure S1.6d shows, the extremely high abundance of rare sequences, and lack of sequences shared among a majority of sites, does not support an analysis of occupancy, and hence this study focused on the substantial diversity observable at the genus and family levels.

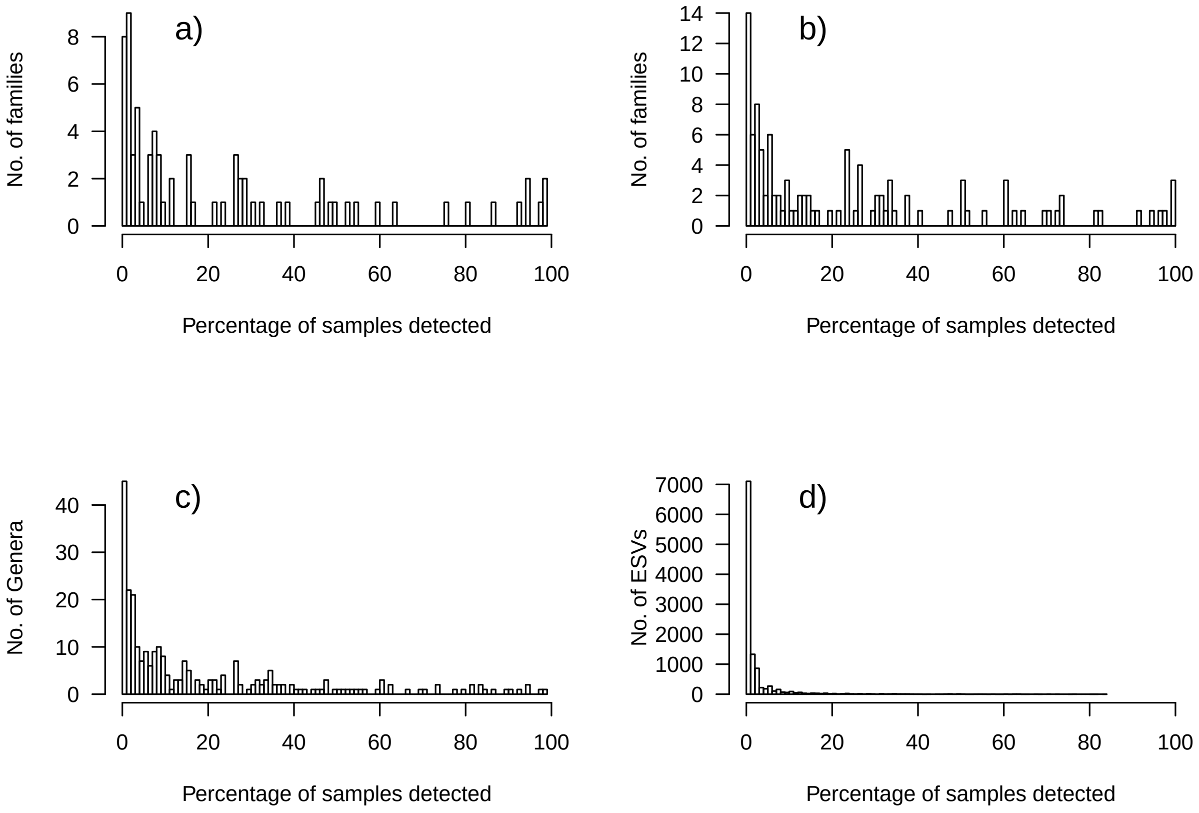

Figure S1.6: Prevalence of taxonomic units in the 126 (a) and 138 (b,c,d) samples processed using CABIN or DNA metabarcoding. Panel a) are morphologically identified taxa, predominantly at the family-level, b) metabarcoding at the family-level, c) metabarcoding at the genus-level, and d) Exact Sequence Variants (ESV).

Exploration of community data

Firstly, looking at the metabarcoding data at the rank of genera, we found that compositional turnover among replicate samples from the same site, at the same time, and using the same primer, was on average greater than 30%:

Table S1.3: Mean genus-level dissimilarity of replicate samples collected from the same site, and during the same visit, as identified by DNA metabarcoding using two sets of primers.

	PAD1	PAD3	PAD4	PAD11	PAD14	PAD33	PAD37	PAD38	Mean
BR5	0.27	0.26	0.34	0.27	0.31	0.42	0.32	0.36	0.32
F230	0.34	0.30	0.34	0.28	0.29	0.35	0.28	0.33	0.31

Secondly, turnover was typically greater than 50% among sites from the same survey period (again using the same primer).

Table S1.4: Mean genus-level dissimilarity of samples collected from different sites within the Peace-Athabasca Delta, during the same visit (same season and year), as identified by DNA metabarcoding using two sets of primers.

	PAD1	PAD3	PAD4	PAD11	PAD14	PAD33	PAD37	PAD38	Mean
BR5	0.54	0.50	0.56	0.54	0.54	0.50	0.52	0.54	0.53
F230	0.52	0.48	0.51	0.51	0.50	0.47	0.48	0.50	0.50

Finally, we could expect compositional dissimilarity to gradually increase over time before reaching a plateau over longer timescales. However, relative to the rapid turnover observed among replicate samples, and among sites, dissimilarity of samples from the same location separated in time remained relatively uniform for most sites. We also did not find any evidence to suggest that taxa were any more likely to be detected at the same site in subsequent years than random, given their prevalence in our dataset.
Note that although there appeared to be a sharp increase in dissimilarity after 5 years, we only had a single pairwise comparison per site over this length of time, whereas many more combinations exist for samples taken 1-3 years apart.

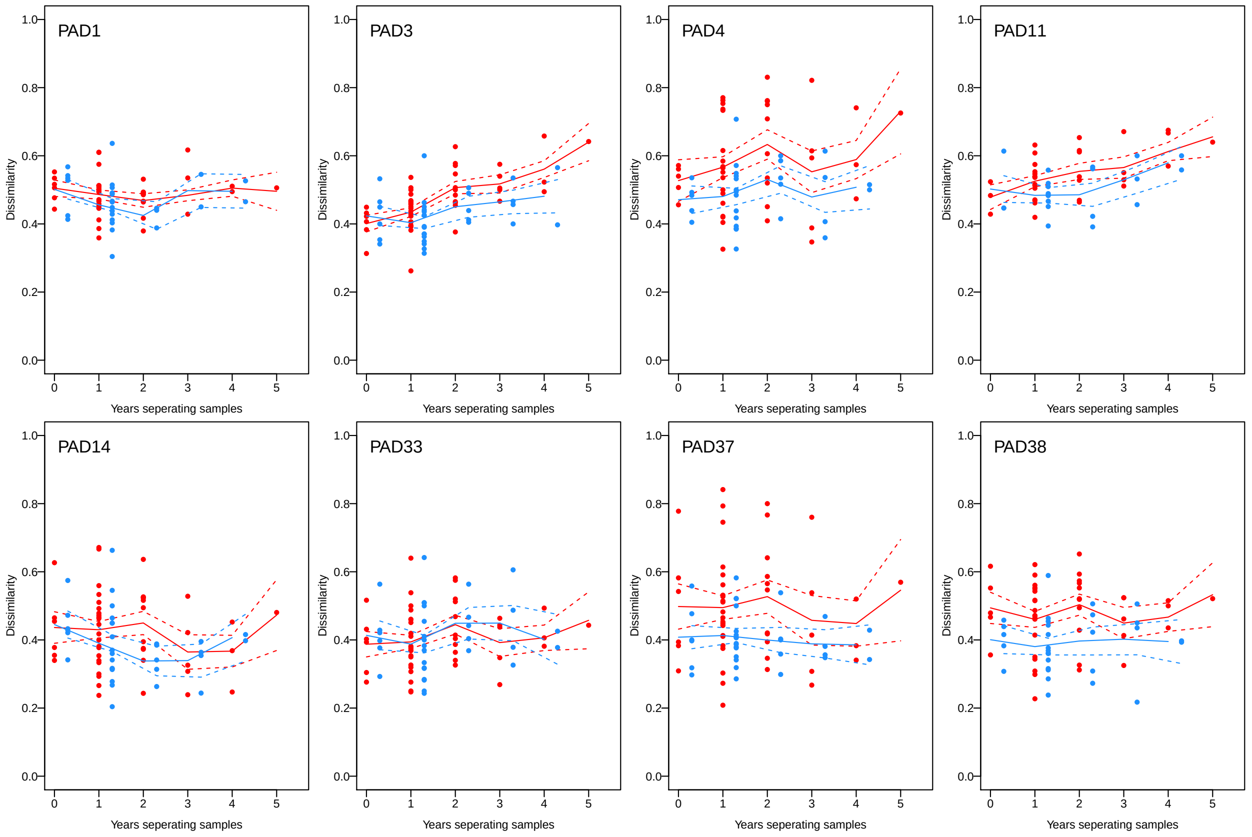

Figure S1.7: Relationship between invertebrate pairwise dissimilarity (Sorenson index) from the same site location given their seperation in years. See Table S1.1 for locations based on site numbers. Dissimilarity was calculated for both BR5 (red) and F230 composition (blue), and lines indicate the mean and 95% confidence intervals of a polynomial regression.

Hierarchical Model Framework

Occupancy Modelling

Hierarchical models recognize that due to imperfect detection, biodiversity survey data is not a record of species' presence and absence at a list of sites, but rather a list of occasions in which a species was detected or not detected. To accounts for that error, a hierarchical model estimates two parallel processes, the state process that describe's a species distribution or abundance, and the detection, or observation, process. Additional information is needed to describe the detection model and this is available when repeated separate observations (in our case benthic invertebrate samples) are taken over a short period of time. Long periods cannot elapse between samples because we assume the system remains stable, and the samples are comparable to one another. Many other study designs are possible to generate similar information on detection [14]. If we assume the likelihood of false-positives is negligible, then detections and non-detections from repeated observations provide information to the model about how likely a species is to be missed by any one particular sample. The model can then be extended in a variety of ways to account for factors influencing the observation process (such as our choice of primers in metabarcoding), and factors influencing the state process, namely the true distribution of species.

Within occupancy models, fitted to records of detection/non-detection, observations are described as an outcome of independent Bernoulli trials to estimate the probability of detecting a species at occupied

sites. Conversely an N-mixture model estimates the latent abundance of each taxon, rather than just their occurrence, as a function of a Poisson distribution. In both cases the single-species format is extended to model communities by assuming that species' responses to environmental conditions are similar and can therefore be modelled as random effects within a common distribution. The hyper-parameters that describe this distribution can be estimated as another tier in the hierarchical model [15]. This study followed the framework and model structure outlined by Kery and Royle [16]. Our code is provided below to encourage reeaders to adopt and modify it for their needs, but we urge interested readers to consult Kery and Royle [16] for further information.

As described in the main text, four models were constructed to represent the various data types avaiable:
1. *CABIN Fcount*: CABIN counts of families
1. *CABIN Fpa*: CABIN presence-absence of families
1. *DNA Fpa*: DNA presence-absence of families
1. *DNA Gpa*: DNA presence-absence of genera

In each case we tested whether the state-process (i.e. true occupancy/abundance) was influenced by seven environmental covariates (four hydrological and three temperature-based). The observation-process model (i.e. detectability) for *DNA-Fpa* and *DNA-Gpa* was split according to which primer-pair detected a given taxon. Finally, we tested whether detectability responded to the proportion of the sample that had been processed (*CABIN Fpa* model), or the total sequencing depth (*DNA Fpa* & *DNA Gpa* models). We expected greater "effort" during sample processing would have increased the total number of taxa identified. This expectation has been supported by studies of both traditional biomonitoring (e.g. [17] and references therein), and DNA metabarcoding (e.g. [18]). However, covariates for processing effort were not retained in any of the final occupancy models. Given we are aware greater processing effort increases detectability, we suspect that on this occasion, the effect of processing covariates on detection were too subtle, relative to other differences such as primer bias, and the substantial overall turnover in our system. Note the proportion of the sample that had been processed was retained as a covariate in the N-mixture model.

Detection Matrix
The community composition matrices were formatted as arrays for sites, replicates and taxa. The dimension *sites* refers to the 62 unique combinations of 8 sites, 1 or 2 seasons, and 6 years. The *replicates* dimension includes 3-6 columns based on a maximum number of attempts to observe the composition of the community during site visits. For CABIN sampling, triplicate samples were equivalent to 3 observations, but for metabarcoding, the processing of samples twice with separate pairs of primers represents independent observations of the community, and hence up to 6 replicate observations. Detection of all *taxa* is then reflected in each layer of the array, namely 74 and 109 at family-level for CABIN and metabarcoding respectively, and 263 at the genus-level for metabarcoding. Note that where there was no survey e.g. because we did not survey all sites in triplicate every year, the entries in the detection matrix were set as NA's.

Data Augmentation
To extend the model infernce to encompass taxa that were never detected requires a process known as data-augmentation (see [19], and more recently [20]). The key addition to the basic occupancy model structure is an overarching process that determines how many taxa from a larger superpopulation M are expected to be included in the system. The fraction of M that are included is described by Ω. Ideally the parameter M would be informed by some prior knowledge of how many species were present in the broader region but given we do not know this, we chose a vague prior, large enough that the posterior distribution of Ω was not close to 1, but also not so high that the model took far longer to converge.

Thus, the detection matrix was expanded to allow for data augmentation. See page 682 of [16] for more details:

```
# Total number of Genera identified by metabarcoding
nspec
# 263
# Vague prior on Ntotal: M = approximately 50% more than nspec
nz   = (400-nspec)
# Empty array with only zeroes
```

```
Yaug = array(0, dim=c(nsite, nrep, nspec+nz))
# Copy into it the observed data
Yaug[,,1:nspec] = Y
# Now create same NA pattern in the augmented matrix, as was the case for the observed taxa.
missings = is.na(Yaug[,,1])                          # e.g. no replicates in 2016
for(k in (nspec+1):(nspec+nz)){ Yaug[,,k][missings] = NA }
```

JAGS Models

The code below provides the structure for the occupancy-detection model, with numbers taken from the DNA metabarcoded genus-level model, but the format was the same for the family-level models too. For illustration, the code includes indicator variables, which are used to estimate the importance of a given covariate in model fitting, and although the starting model included 7 possible covariates, the code below has been reduced to three.

A reminder of the inputs:

```
# Bundle and summarize data
win.data = list(Y       = Yaug,             # Response data
                nsite   = dim(Y)[1],        # No. of sites
                nrep    = dim(Y)[2],        # Max no. of replicates
                nspec   = dim(Y)[3],        # No. of observed species
                nz      = nz,               # No. of hypothetical species
                M       = nspec + nz,       # Metacommunity richness
                X       = X,                # Environmental covariates
                ncov    = ncol(X)           # No. of covariates
)

# Specify model in BUGS language
sink("PAD_Gen_Occ_model.txt")
cat("
    model {

    # Data Augmentation priors - Fraction of superpopulation present in this community
    omega ~ dunif(0,1)

    # Priors for species-specific effects in occupancy and detection
    for(k in 1:M){
      # Occupancy intercept
      lpsi[k]       ~ dnorm(mu.lpsi, tau.lpsi)
      # Occupancy response slope
      for(i in 1:ncov){
        betalpsi[k,i]   ~ dnorm(mu.betalpsi[i], tau.betalpsi[i])
      }
      # Detectability (seperate estimate per primer pair)
      for(j in 1:2){
        lp[k,j]         ~ dnorm(mu.lp[j], tau.lp[j])
      }
    }

    # Hyperpriors
```

```
# For the model of occupancy
mu.lpsi        ~ dnorm(0,0.1)                # Community mean of occupancy
tau.lpsi       = pow(sd.lpsi, -2)
sd.lpsi        ~ dunif(0,8)                  # Standard deviation of occupancy

for(i in 1:ncov){
  mu.betalpsi[i]    ~ dnorm(0,0.1)           # Occupancy response to each covariate
  tau.betalpsi[i]   = pow(sd.betalpsi[i], -2)
  sd.betalpsi[i]    ~ dunif(0, 4)            # Standard deviation of coefficent
}

# Indicator variables (used when selecting which covariates to retain).
for(i in 1:ncov){
  V[i] ~ dbern(0.5)
}

# Community mean(s) of detection (logit) divided by the type of Primer
for(j in 1:2){
  mu.lp[j]          ~ dnorm(0,0.1)
  tau.lp[j]         = pow(sd.lp[j], -2)
  sd.lp[j]          ~ dunif(0, 4)
}

# Data Augmentation process: Ntotal taxa sampled out of M available
for(k in 1:M){
  w[k] ~ dbern(omega)
}

# Ecological model for true occurrence (process model)
for(k in 1:M){
  for(i in 1:nsite) {
    logit(psi[i,k]) = lpsi[k] +
                      (betalpsi[k,1] * X[i,1] * V[1]) +
                      (betalpsi[k,2] * X[i,2] * V[2]) +
                      (betalpsi[k,3] * X[i,3] * V[3])
    mu.psi[i,k]     = w[k] * psi[i,k]
    z[i,k]          ~ dbern(mu.psi[i,k])
  }
}

# Observation model for replicated detection/nondetection observations
for(k in 1:M){
  for(i in 1:nsite){
    for(j in 1:nrep){
      logit(p[i,j,k]) = lp[k,PRI[j]]
      mu.p[i,j,k]     = z[i,k] * p[i,j,k] * step(anyseq[i,j]-1)
      Y[i,j,k]        ~ dbern(mu.p[i,j,k])
    }
  }
}

# Derived quantities
for (i in 1:nsite){ Nsite[i] = sum(z[i,]) }     # Number of species at each site
```

```
    n0      = sum(w[(nspec+1):(nspec+nz)])          # Number of unseen species
    Ntotal  = sum(w[])                              # Total metacommunity size

    # Vectors to save (capital S in name is for 'save')
    # To save space, discard samples for all except 1 of the potential species.
    lpsiS[1:(nspec+1)]            = lpsi[1:(nspec+1)]
    betalpsiS[1:(nspec+1),1:ncov] = betalpsi[1:(nspec+1),1:ncov]
    lpS[1:(nspec+1),1:2]          = lp[1:(nspec+1),1:2]

    }
    ",fill = TRUE)
sink()

# Initial values
wst = rep(1, nspec+nz)                    # Start by including every taxon
zst = array(1, dim = c(nsite, nspec+nz)) # Set every taxon to start as occurring
inits = function() list(z         = zst,
                        w         = wst,
                        lpsi      = rnorm(n = nspec+nz, sd=0.1),
                        betalpsi  = matrix(rnorm((nspec+nz)*ncol(X), sd=0.1),
                                           nrow=nspec+nz, ncol=ncol(X) ),
                        lp        = matrix(rnorm(n = (nspec+nz)*2, sd=0.1),(nspec+nz),2),
                        V         = rep(1,ncol(X)) )

# Set 1
params1 = c("omega", "mu.lpsi", "sd.lpsi", "mu.betalpsi", "sd.betalpsi",
            "mu.lp", "sd.lp", "Ntotal", "Nsite", "lpsiS", "betalpsiS","lpS","V")

# MCMC settings
ni = 1000000
nt = 50
nb = 250000
nc = 4

# Run JAGS with indicator variables
mod = jags(data = win.data,
           inits,
           parameters.to.save = params1,
           model.file = "PAD_Gen_Occ_model.txt",
           n.chains   = nc,
           n.thin     = nt,
           n.iter     = ni,
           n.burnin   = nb,
           parallel   = FALSE,
           codaOnly   = TRUE,
           verbose    = TRUE)
```

Convergence, model fit and variable selection

The most complex models included hundreds of taxa, data-augmentation and multiple-covariates, and hence convergence of all parameters was slow, evaluated based on their *Rhat* values [21]. Updating initial values

with previous runs did reduce run time, but ultimately some coefficients, particularly for some rare species, never fully converged. Recently, Warton et al. [22] described how commonly used measures of model fit, particularly the Pearson chi-square goodness-of-fit statistic and Bayesian P-value [23,24] were not suitable for assessing incidence-based occupancy models. We therefore followed their approach and looked at the diagnostic plots based on Dunn-Smyth residuals to assess whether different variable combinations could improve model fit.

To select which variables contribute to the occupancy model most effectively we used the latent indicator variable approach [25]. Latent variable indicators were particularly slow to converge, but to allow for potential interactions, we followed a process of backwards selection, eliminating variables if there was clear evidence they were rarely included in the fitted models once they had converged (i.e. posterior distribution of v < 0.5).

N-mixture Model

Occupancy models are incidence-based, modelling the detection/non-detection of each taxon. This is in part because the sequence data generated by metabarcoding is typically not regarded as quantitative, and we therefore do not have estimates of relative abundance ([25] and references therein). Conversely the processing of CABIN samples includes counts of each taxon observed in a subset of the whole sample. Given the impact of environmental changes on the aquatic invertebrate community are reflected in changes to taxon abundances, a separate "abundance-based" model, known as N-mixture modelling, was applied to the CABIN samples [27] to incorporate as much of the information present in CABIN samples as possible.

The underlying process of an N-mixture model assumes abundances follow a Poisson distribution, but the model structure is otherwise similar to that of the incidence-based occupancy model. The proportion of the sample processed was retained as a covariate (*alpha*) on detectability in the N-mixture model (i.e. taxon detectability and abundance was dependent on the proportion of a sample that is sorted). Note that the N-mixture model estimates the probability a given number of **individuals** were present in a sample, and the probability of detection was also estimated at the individual-level. As a result we cannot easily compare between the occupancy and N-mixture models without making some assumptions about how many individuals we can count at a site. Therefore, the intention of the N-mixture model was to compare the broad distribution of occurrence vs. detectability (Fig. S10), and the relative influence of environmental factors on those distributions.

```
# Standardize the proportion of a sample processed
# Typically 5% but ranges from 3% to 100% of the sample collected.
sort.count = standardize(perc_sorted)
# Split into site-x-replicate matrix
scount = matrix(0,ncol=3,nrow=dim(YC)[1])
for(i in 1:length(scount)){
  scount[ match( sample.list[i], dimnames(YC)[[1]] ),
          match( rep.list[i],    dimnames(YC)[[2]]) ] = sort.count[i]
}

#-x-x-x-x-x-x-x-x-x-x-x-x-x-x-x-x-x-x-x-x-x-x-#

# N-mixture model in BUGS language
cat("
    model {

    # Data Augmentation priors - Fraction of superpopulation present in this community
    omega ~ dunif(0,1)

    # Priors for species-specific effects in occupancy and detection
```

```
for(k in 1:M){
  # Occupancy intercept
  lpsi[k]  ~ dnorm(mu.lpsi, tau.lpsi)
  # Occupancy response slope
  for(i in 1:ncov){ betalpsi[k,i] ~ dnorm(mu.betalpsi[i], tau.betalpsi[i]) }
  # Detection intercepts
  lp[k]         ~ dnorm(mu.lp, tau.lp)
  # Slope of detection
  alpha[k]      ~ dnorm(mu.alpha, tau.alpha)
}

# Hyperpriors

# For the model of occupancy
mu.lpsi    ~ dnorm(0,0.01)                    # Community mean of occupancy
tau.lpsi   = pow(sd.lpsi, -2)
sd.lpsi    ~ dunif(0,8)                       # Standard deviation of occupancy

for(i in 1:ncov){
  mu.betalpsi[i]    ~ dnorm(0,0.1)            # Occupancy response to each covariate
  tau.betalpsi[i]   = pow(sd.betalpsi[i], -2)
  sd.betalpsi[i]    ~ dunif(0, 4)             # Standard deviation of coefficent
}

# Indicator variables (used when selecting which covariates to retain).
for(i in 1:ncov){
  V[i] ~ dbern(0.5)
}

# Community mean(s) of detection (logit) divided by the type of Primer
mu.lp           ~ dnorm(0,0.1)                # Community mean of detection
tau.lp          = pow(sd.lp, -2)
sd.lp           ~ dunif(0, 4)

mu.alpha        ~ dunif(-0.5,0.5)             # Mean coefficient upon detection
tau.alpha       = pow(sd.alpha, -2)
sd.alpha        ~ dunif(0, 4)

# Data Augmentation process: Ntotal species sampled out of M available
for(k in 1:M){
  w[k]          ~ dbern(omega)
}

# Ecological model for true occurrence (process model)
for(k in 1:M){
  for(i in 1:nsite) {
    N[i,k]      ~ dpois(lambda[i,k] )* w[k])
    log(lambda[i,k]) = lpsi[k] +
                       (betalpsi[k,1] * X[i,1] * V[1]) +
                       (betalpsi[k,2] * X[i,2] * V[2]) +
                       (betalpsi[k,3] * X[i,3] * V[3])
  }
}
```

```
    # Observation model for replicated detection/nondetection observations
    for(k in 1:M){
      for(i in 1:nsite){
        for(j in 1:nrep){
          logit(p[i,j,k]) = lp[k] + (alpha[k] * scount[i,j]) # step(anycount[i,j]-1)
          YC[i,j,k] ~ dbin(p[i,j,k], N[i,k])
        }
      }
    }


}"
```

Results

Metacommunity size (gamma diversity)

Given the probability of detecting the taxa we observed in each dataset, data augmentation suggests how many more taxa are likely to be present in the metacommunity, but have not been observed so far. The models suggest that another ~20 families, or 100 genera, may potentially occur in the PAD system that have not been detected. Note that while data augmentation was present in each model, and hence will have to some extent influenced the overarching distribution of parameters at the community-level, the results for the augmented taxa were not stored after the model run. As a result, all the results we present below are solely for the detected taxa.

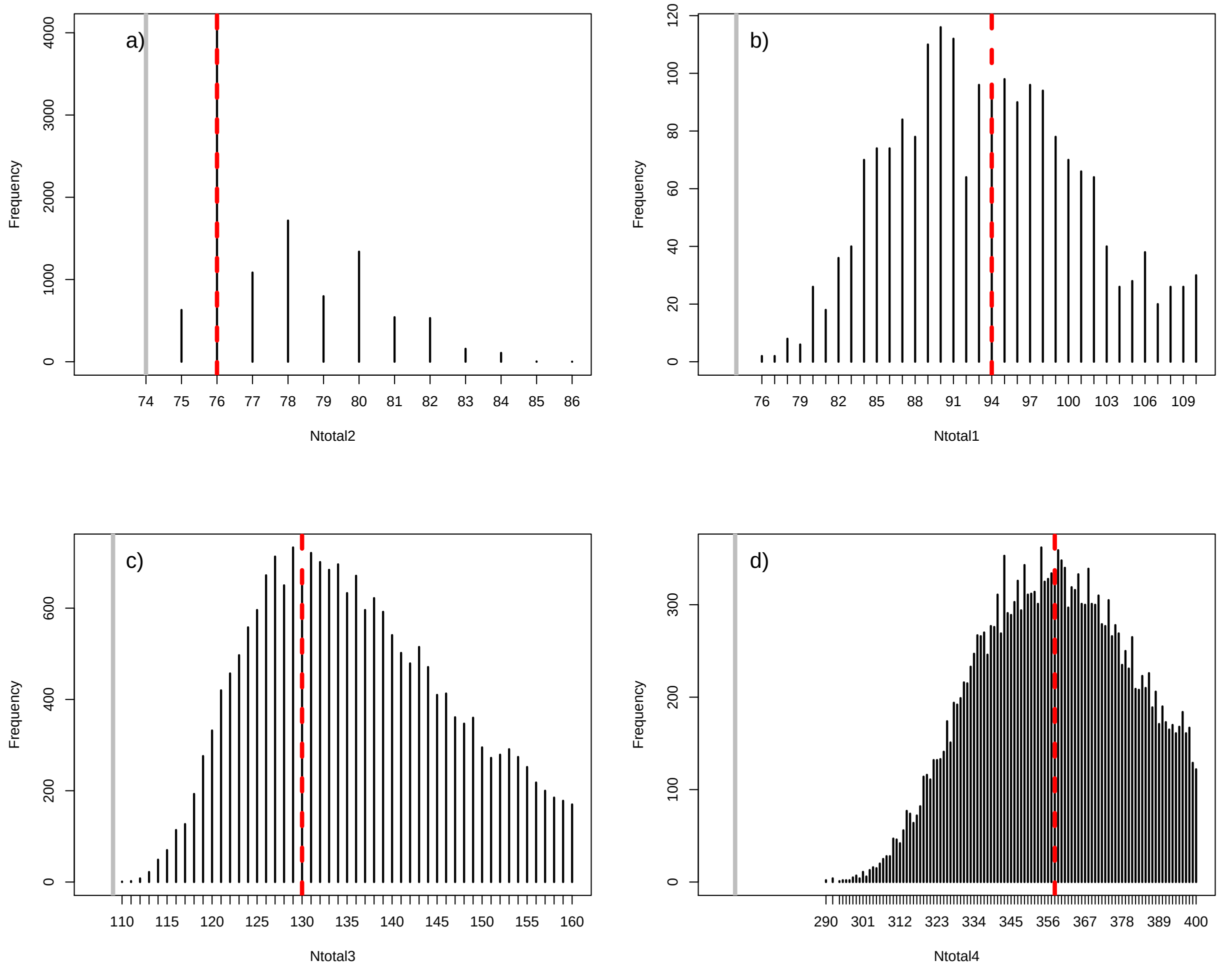

Figure S1.8: Estimates of the total number of taxa in the PAD metacommunity based on a) CABIN count data, b) CABIN presence/absence data, and metabarcoding data at c) family and d) genus level. Grey bars indicate the observed number of taxa, and the red dashed lines marks the mean model estimate after accounting for imperfect detection.

Site richness (alpha diversity)

As with the metacommunity, given the probability of detecting taxa is <1, the true richness of each sampling site is likely to be greater than what was actually observed given the sampling effort expended. The degree to which richness is underestimated varies among each model, but in every case, the true number will be greater than was observed, and Figure S1.9 simply illustrates this for the 8 sites sampled in 2016.

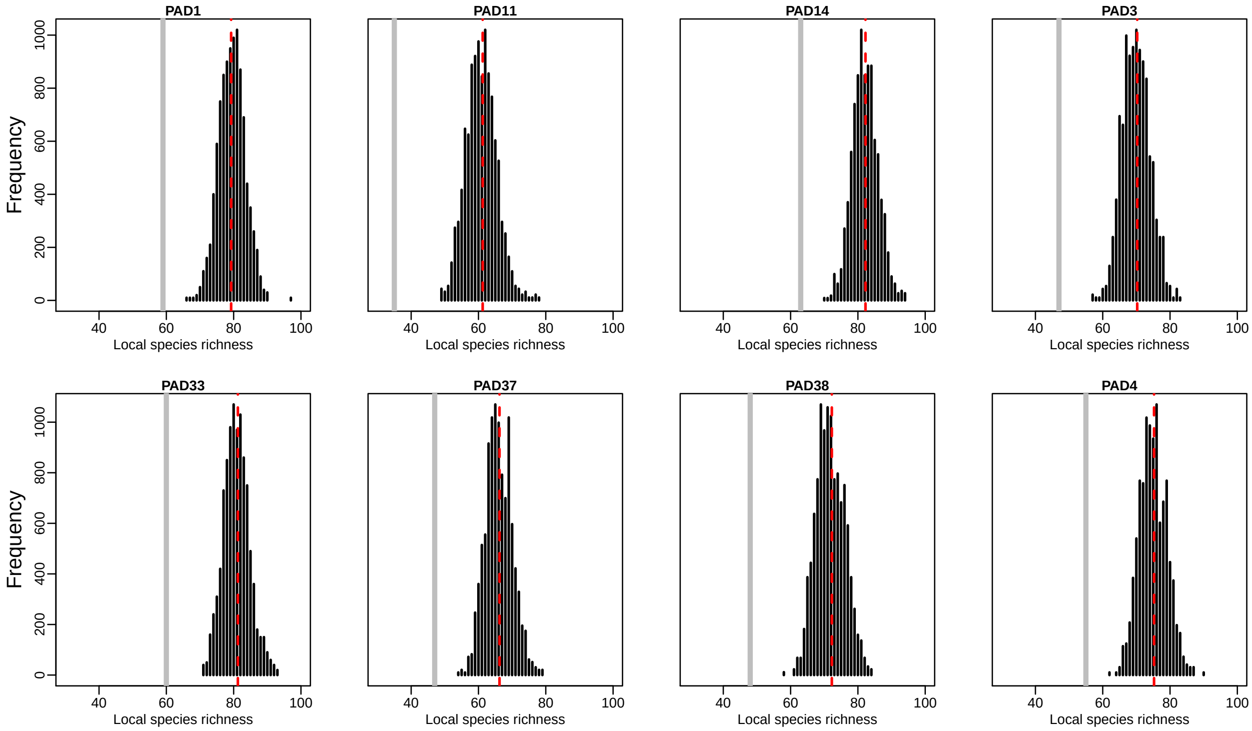

Figure S1.9: Estimated number of genera in each of the eight field sites during August 2016 predicted by the occupancy model trained on metabarcoding data. The grey line indicates the number of genera directly detected, and the red line marks the mean model estimate after accounting for imperfect detection.

Occupancy and detectability

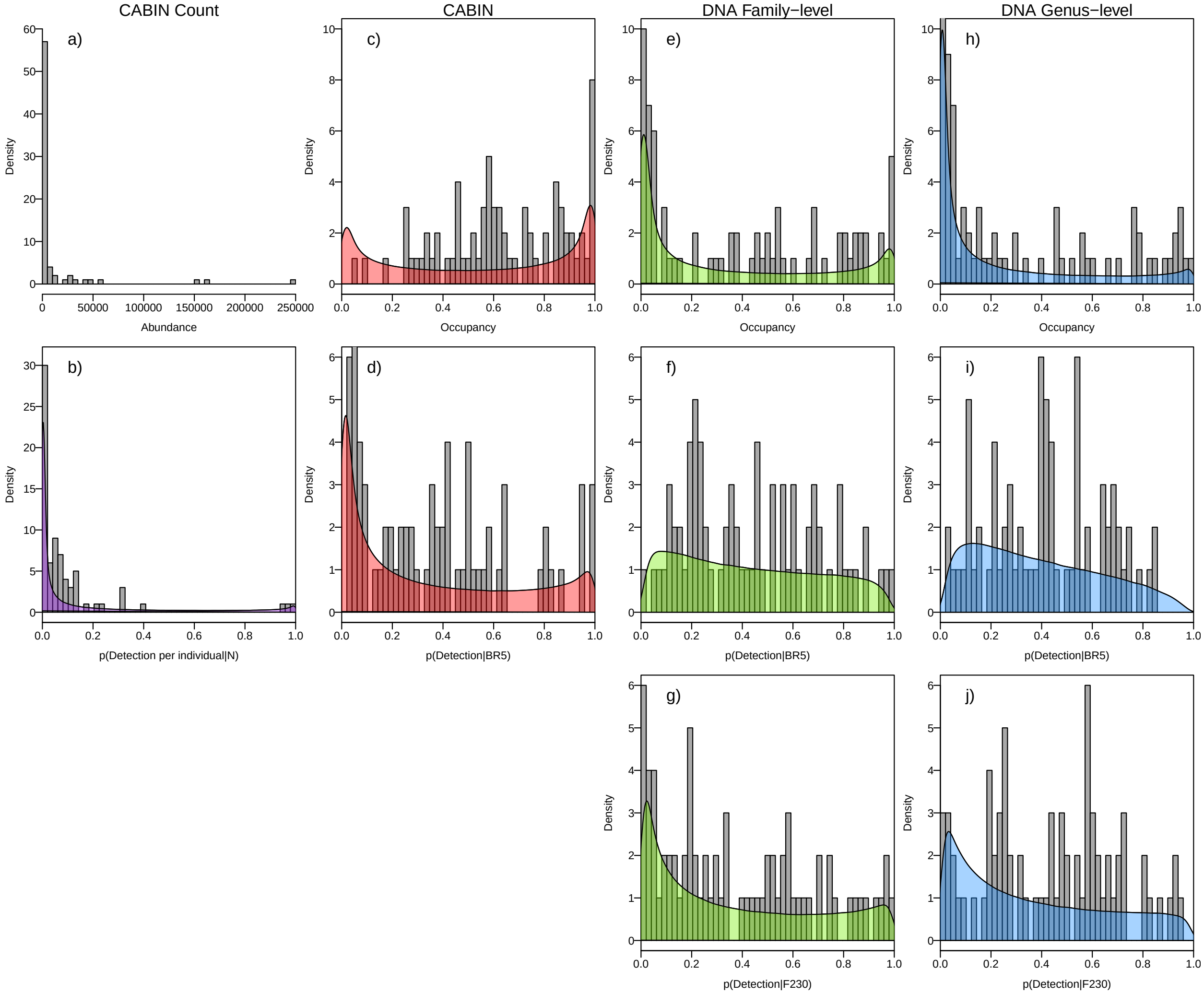

Figure S1.10: This figure expands upon Figure 2 in the main text to include the modelled distribution of occupancy and detectability for the CABIN N-mixture model of abundance and metabarcoding model at the genus-level. Columns show the predicted abundance and *individual* detectability based on CABIN count data (a,b), and then taxon occupancy and detectability based on CABIN presence-absence data (c,d), metabarcoding at family-level (e-g), and metabarcoding at the genus-level (h-j). Detectability of taxa with metabarcoding is subdivided into the primer pairs used by this study. The shaded polygons describe the probability density of the community-level hyperparameters, and the grey bars indicate the underlying frequency of the parameters estimated for each taxon individually.

CABIN vs. metabarcoding at family-level

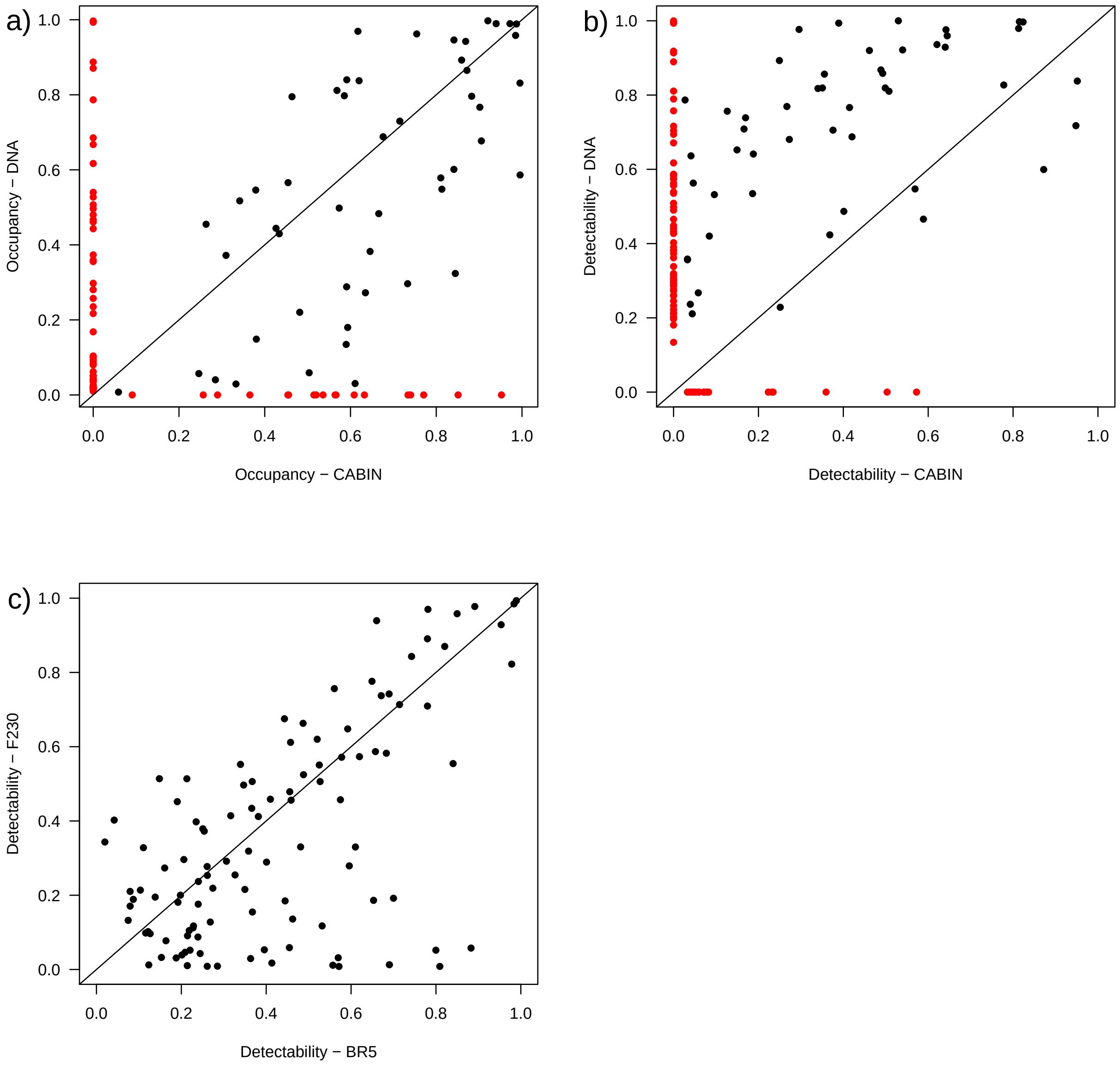

Figure S1.11: Comparison of a) occupancy and b) detectability estimates in models trained by CABIN data and DNA metabarcoded data at the family-level (n= 50). Red points indicate the taxa not observed by the complementary method i.e. 18 and 59 families were unique to CABIN and metabarcoding respectively. See below for further information on the identity of unique taxa. Panel c) breaks down the detectability of families detected using within DNA metabarcoding (y-axis in panel b) for each of the two primers used.

Taxa unique to one approach

Before proceeding with any comparisons, we highlight the discrepancies in the taxonomic lists between the CABIN (morphological) and metabarcoding datasets. Note that without a control sample to validate either

approach (i.e. a mock community of known composition), there are several reasons why taxa may be missed by one approach or the other, and the reasons may differ among taxa. Nonetheless, we show where such discrepancies exist in our data, and explore some plausible reasons for those. We refer readers to previous papers [26,28] for more discussion of comparisons between morphological and metabarcoding approaches.

Table S1.5 summarises the taxa identified by morphology in the CABIN samples, but absent from the metabarcoding dataset, and Table S1.6 provides the equivalent for taxa only identified by metabarcoding. To try to understand why some taxa might have been missed by metabarcoding, we have also included columns in Table S1.5 describing how often the taxa were detected, and the number of reference sequences available in the DNA barcode library. The first point is that the majority of taxa in Table 5 are aquatic mites, which are challenging to identify reliably, and as a result are poorly represented in DNA barcode libraries. Other taxa like Dixidae are also poorly represented in the reference library and hence the bioinformatic pipeline was less likely to relate observed sequences to these taxonomic groups. There are reasonably large numbers of reference sequences for Trichoceridae, Perlodidae, Rhyacophilidae, but given these taxa were observed in less than 10% of samples, it is possible they were simply absent from the samples processed by metabarcoding, or at such low biomass that they were less likely to be detected (see references above). Therefore, the area we would suggest is most interesting is the absence of Chaoboridae and Sminthuridae, both of which are relatively prevalent and have reference sequences (see [29]). The specimens from the PAD may have been incorrectly identified, or reference sequences are from species within the same taxonomic group, but relatively unrelated to the species in the PAD, making assignment less certain.

Table S1.5: Taxonomic classification of taxa that were observed in the CABIN morphological dataset, but never observed using DNA metabarcoding. The number of reference sequences describe the coverage of each family within the library used by this study (see details in section above on metabarcoding data).

Phylum	Class	Order	Family	No._of_samples_observed	No._of_reference_seqs
Arthropoda	Arachnidae	Trombidiformes	Mideopsidae	1	NA
Arthropoda	Insecta	Trichoptera	Uenoidae	1	67
Arthropoda	Insecta	Diptera	Trichoceridae	1	153
Arthropoda	Arachnidae	Trombidiformes	Eylaidae	2	NA
Arthropoda	Arachnidae	Trombidiformes	Sperchontidae	2	NA
Arthropoda	Arachnidae	Trombidiformes	Torrenticolidae	2	NA
Mollusca	Gastropoda	Basommatophora	Ancylidae	2	48
Arthropoda	Insecta	Plecoptera	Perlodidae	3	271
Arthropoda	Arachnidae	Trombidiformes	Hydryphantidae	4	3
Arthropoda	Insecta	Hemiptera	Mesoveliidae	5	52
Arthropoda	Insecta	Trichoptera	Rhyacophilidae	5	837
Arthropoda	Insecta	Diptera	Dixidae	7	3
Arthropoda	Arachnidae	Trombidiformes	Hygrobatidae	10	144
Arthropoda	Arachnidae	Trombidiformes	Oxidae	11	NA
Arthropoda	Arachnidae	Trombidiformes	Hydrodromidae	14	NA
Arthropoda	Insecta	Diptera	Chaoboridae	45	95
Arthropoda	Collembola	Collembola	Sminthuridae	61	201

Table S1.6: Families of aquatic invertebrates included in this study that were detected using DNA metabarcoding, but were otherwise not observed using standard morphological approaches, and the percentage of samples in which they were detected using each primer set.

Taxon	BR5_percent	F230_percent	______	Taxon	BR5_percent	F230_percent
Apatelodidae	0.0	0.7	–|–	Isonychiidae	0.7	0.0
Asplanchnidae	13.9	0.0	–|–	Lecanidae	6.6	5.1
Belostomatidae	1.5	0.7	–|–	Lepadellidae	8.8	0.0
Bosminidae	4.4	0.0	–|–	Leptodoridae	0.7	0.0

Taxon	BR5_percent	F230_percent	______	Taxon	BR5_percent	F230_percent
Brachionidae	3.6	2.9	–|–	Limoniidae	2.2	2.9
Brachyceridae	2.2	6.6	–|–	Lubomirskiidae	1.5	14.6
Caeciliusidae	2.2	2.2	–|–	Lumbricidae	0.0	0.7
Candonidae	16.8	25.5	–|–	Lumbriculidae	45.3	42.3
Chaetonotidae	0.7	2.9	–|–	Metropodidae	0.7	0.7
Chloropidae	2.9	1.5	–|–	Muscidae	0.7	0.0
Chrysopidae	0.7	0.0	–|–	Noctuidae	0.7	1.5
Chydoridae	59.1	13.1	–|–	Perlidae	1.5	0.0
Cicadellidae	3.6	0.0	–|–	Plumatellidae	18.2	10.9
Corycaeidae	0.7	0.0	–|–	Poduridae	2.2	5.1
Cyclopidae	26.3	0.0	–|–	Polyphemidae	34.3	0.0
Cyprididae	78.1	81.0	–|–	Scathophagidae	2.9	2.9
Daphniidae	99.3	69.3	–|–	Scirtidae	2.9	4.4
Diaptomidae	54.0	51.8	–|–	Sialidae	0.7	0.0
Dolichopodidae	3.6	1.5	–|–	Sididae	26.3	5.1
Elmidae	1.5	0.0	–|–	Simuliidae	7.3	0.7
Enchytraeidae	0.7	0.0	–|–	Sisyridae	2.2	5.8
Erpobdellidae	19.0	13.9	–|–	Spongillidae	11.7	0.0
Eulophidae	0.7	0.7	–|–	Stenostomidae	13.1	0.0
Eurycercidae	74.5	4.4	–|–	Synchaetidae	0.7	0.7
Glossiphoniidae	13.9	29.2	–|–	Tabanidae	1.5	2.9
Helophoridae	0.7	0.7	–|–	Trichocercidae	2.2	0.0
Hydraenidae	0.7	1.5	–|–	Tubificidae	100.0	82.5
Hydropsychidae	3.6	0.0	–|–	Ulidiidae	0.7	0.0
Hypsibiidae	13.9	9.5	–|–	NA	NA	NA

Family-level vs genus-level

Figure S1.12 compares the estimates of occupancy and detectability derived from the *DNA Fpa* and *DNA Gpa* models. The majority of genera are less prevalent than their parent ranks. Naturally single-genus families are as common and hence lie on the 1:1 line, but for groups with multiple genera, occupancy could be much lower (e.g. see Chironnomidae in red), and those differences in occupancy may reveal difference sin environmental preferences and potentially competitive interactions. If the most common genus in a family (e.g. Culicidae and Glossiphonidae) was not as common as the parent-family, the distributions of genera were at least partially non-overlapping, and hence there may be evidence to suggest these differences are the reuslt of niche differences or competition. Likewise, rare genera may coexist with more common members of the family, but their narrow range may again be the result of niche differences.
With respect to detectability, we would expect specific genera to be more difficult to observe than families simply because they are less abundant than all members of the family combined. Nonetheless, there are instances when detectability of a specific genus is actually much higher than at the family-level because estimates were averaged for other genera. Note that although genera cannot be more common than their parent-families, estimates of occupancy are conditional on the likelihood of detection, and in some cases the *DNA Gpa* predicted occupacy marginally higher than that of the *DNA Fpa*. This is likely to occur if a genus was observed in many locations, and was actually quite hard to detect.

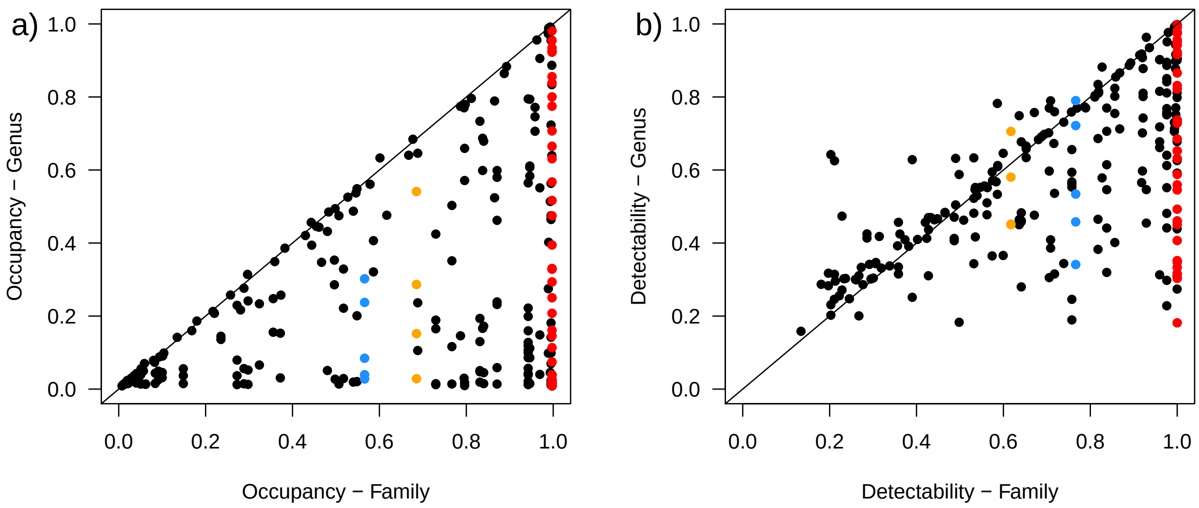

Figure S1.12: Comparison of a) occupancy and b) detectability between models at the family-level and genus-level. Chironomidae (red), Culicidae (blue) and Glossiphonidae (orange) are highlighted to illustrate the variation among genera.

Environmental effects

Based on the latent indicator variables for the occupancy and N-mixture models based on CABIN data, environmental covariates were included in less than 0.3% of saved iterations.
Occupancy models based on metabarcoding data identified relationships between the occupancy of taxa and environmental factors, but these were generally weak, with the response of most taxa not significantly different from 0. The indicator variables consistently identified the frequency of flooding, the length of time the site had been free of ice, and the maximum temperature that year for inclusion. Figure S1.13 illustrates the specific responses of each genus to each covariate, and the overarching weak effect at the community level. Figure S1.14 further identifies for which taxa these effects were considered significant (i.e. HDI does not overlap 0).

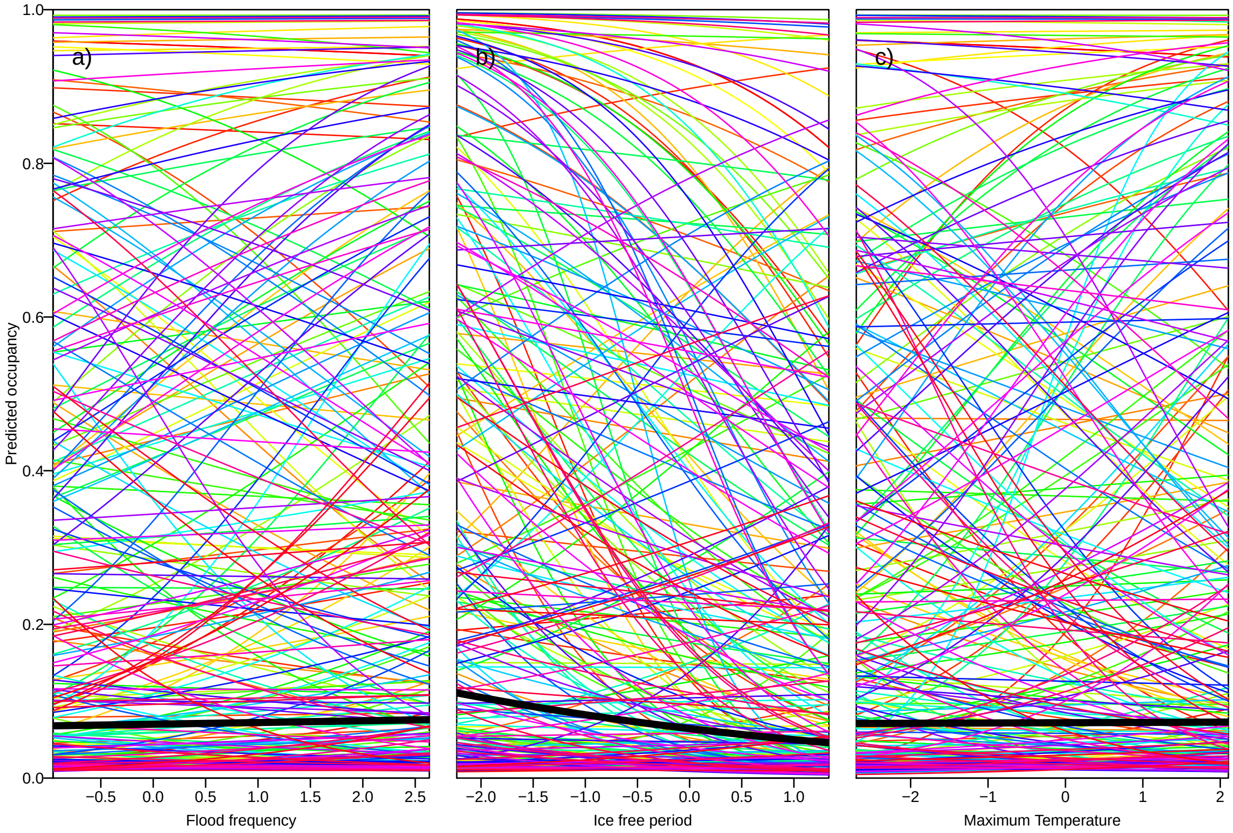

Figure S1.13: Impact on occupancy of specific genera (coloured lines) and the community on average (black line) due to changes in a) site flood frequency, b) the ice free period (prior to survey), and c) maximum temperature (prior to survey that year).

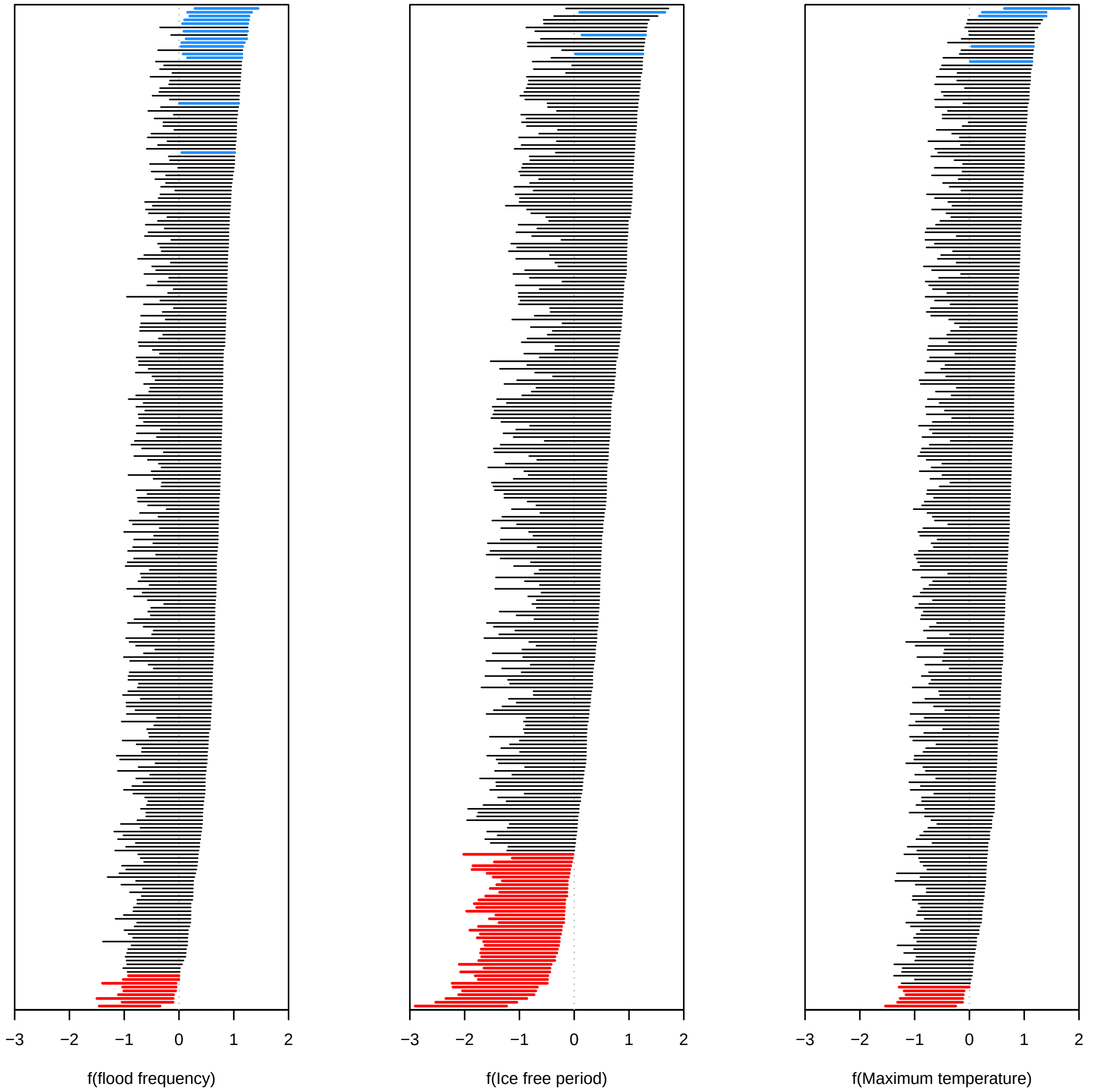

Figure S1.14: Highest density intervals (95%) of coefficients for the three environmental factors with significant positive (blue) and negative (red) effects on the occupancy of genera. Note each panel has been ordered for each coefficient separately and therefore the order of taxa varies in each case.

The genera that responded positively to the frequency of flooding were:

```
## [1] "Angarotipula"   "Banksiola"      "Cloeon"         "Dicrotendipes"
## [5] "Lecane"         "Polyphemus"     "Procladius"     "Triaenodes"
## [9] "Vejdovskyella"
```

The genera that responded negatively to the frequency of flooding were:

```
##  [1] "Acanthodiaptomus" "Carrhydrus"       "Gyrinus"
##  [4] "Laccophilus"      "Limnodrilus"      "Microvelia"
##  [7] "Motobdella"       "Neoporus"         "Phaenopsectra"
## [10] "Piona"            "Plumatella"       "Tubifex"
## [13] "Unionicola"
```

The genera that responded positively to the length of the ice free period were:

```
##  [1] "Aeshna"         "Agabus"         "Aglaodiaptomus" "Anabolia"
##  [5] "Callicorixa"    "Camptocercus"   "Carrhydrus"     "Cladopelma"
##  [9] "Coelambus"      "Corynoneura"    "Culex"          "Daphnia"
## [13] "Dytiscus"       "Eucypris"       "Eurycercus"     "Gerris"
## [17] "Glyptotendipes" "Graphoderus"    "Hesperocorixa"  "Hydaticus"
## [21] "Hydrobius"      "Hygrotus"       "Laccophilus"    "Ladislavella"
## [25] "Lejops"         "Lestes"         "Lumbriculus"    "Microvelia"
## [29] "Motobdella"     "Mytilina"       "Palpomyia"      "Piona"
## [33] "Plumatella"     "Polypedilum"    "Rhantus"        "Sigara"
## [37] "Simocephalus"   "Siphlonurus"    "Sympetrum"      "Tanysphyrus"
## [41] "Thulinius"
```

The genera that responded negatively to the length of the ice free period were:

```
## [1] "Ilybius"     "Nemotaulius" "Phryganea"
```

The genera that responded positively to the maximum temperature prior to survey were:

```
## [1] "Pristina"                    "Sida"
## [3] "Spongilla"                   "Stilobezzia"
## [5] "undef_Chironomidae"          "undef_undef_Sarcoptiformes"
```

The genera that responded negatively to the maximum temperature prior to survey were:

```
## [1] "Aglaodiaptomus" "Agraylea"       "Hedriodiscus"   "Ilybius"
## [5] "Ladislavella"
```

Compositional Turnover (beta diversity)

Given the effect detectability has on the expected 'true' number of species present at a site (alpha diversity) or in the metacommunity (gamma diversity), we would also expect detectability to affect our perception of compositional dissimilarity between sites (beta diversity). Dissimilarity may increase among sites if the majority of taxa that were not detected are also not prevalent, and hence unlikely to be shared among samples. Conversely, dissimilarity may decrease if the taxa that were not detected were in fact relatively common, and hence the probability that two sites do in fact share that taxon increases.
Figure S1.15 illustrates the differences between our original measures of dissimilarity, and the estimates we can now make from the four models based on each dataset, either for inter-annual dissimilarity at a site (left column), or among-site dissimilarity during a single visit (right column). This figure highlights a few key points:
1. Based on the CABIN dataset (a,b,e,f), imperfect detection was leading to an overestimation of pairwise dissimilarity among samples.
2. Based on DNA-metabarcoding (c,d,g,h), estimates from models are similar to the original values, particularly for among-site comparisons. This would suggest that even though large numbers of taxa were missed (see figures S8 & S9), the proportion that are shared or unique to a site remained comparable.
3. Compositional turnover increases as taxonomic resolution increases (c & g vs. d & h).

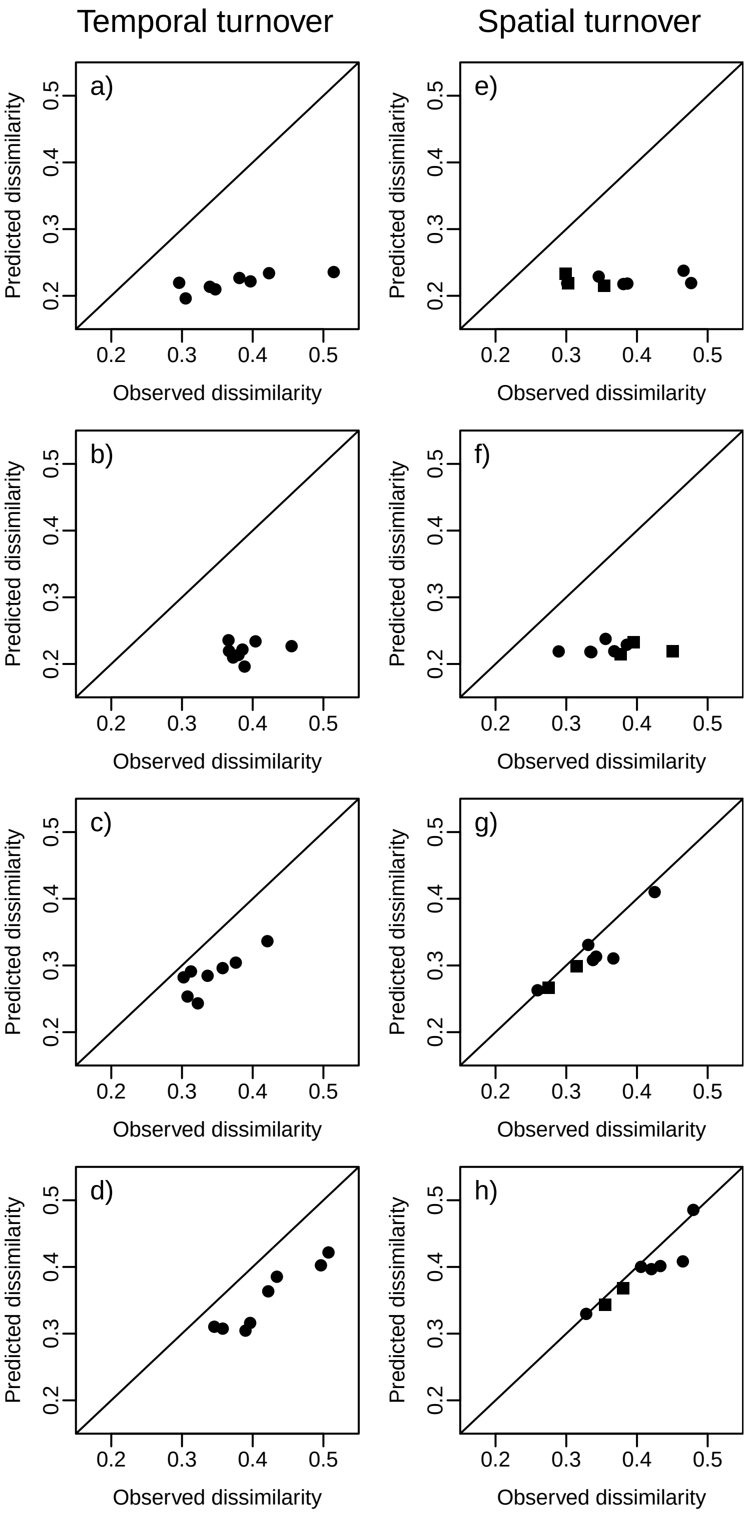

Figure S1.15: Comparison of the mean pairwise dissimilarity (Sorenson) observed in a community and predicted after accounting for detectability. The left column (a-d) compares dissimilarity among samples from the same site in different years, and the right column (e-h) the mean dissimilarity among sites during a single survey (year/season). Rows correspond to the four datasets analysed: CABIN presence-absence data (a,e), CABIN count data (b,f), and DNA metarcoded data at the family (c,g) and genus-level (d,h).

Supplementary Information Appendix 2: Metacommunity simulation and power analysis of macroinvertebrate biomonitoring in the Peace-Athabasca Delta based on morphological identification and DNA metabarcoding.

Alex Bush

26th June 2019

Contents

Power analysis **1**
- The metacommunity . . . 2
- Sensitivity to Impact . . . 5
- The simulation function . . . 6
- Simulation results . . . 12

This supplementary file describes a simulation of our ability to sample and observe change in the aquatic macroinvertebrate metacommunity based on the estimates of diversity, site occupancy and detectability derived from the models described in SI Appendix 1.

Power analysis

The primary aim of biological monitoring is to provide managers with an indication of ecosystem health, alert them to potential degradation of the system, and given most ecosystems are subject to multiple stressors, attribute the most likely cause [30]. Therefore the principle question guiding this research program has been whether significant shifts in the composition of aquatic invertebrates can be detected in the Peace-Athabasca Delta (PAD). We used power analysis to understand how the inherent properties of the PAD ecosystem are likely to affect the quality of the data we can observe, and how reliably changes could be interpreted as statistically significant departures from expectations.

The data properties that influence our statistical power to discriminate between a baseline community and a degraded one are not only dependent on the study design (location and number of samples), but also on the mode of observation, namely the comparison between morphologically-identified CABIN samples and samples processed with DNA metabarcoding. Consequently the analysis below describes how we can understand the relative effect of both sampling effort and processing method on uncertainty in monitoring the aquatic invertebrates of the PAD.

It is worth emphasizing that we do not know the true parameters that describe the aquatic invertebrate metacommunity in the PAD ecosystem. However, by using the hierarchical models described in SI Appendix 1, we do have an estimate of the true *process model* and the *observation model* that governs what data we expect to recover. Therefore the power analysis investigates the sampling required to detect changes to the underlying process model, given our understanding of the observation processes.

To make comparisons based on sampling design and sample processing, we needed to keep the process-model consistent. As such we chose the model at genus-level to be the basis for simulated communities because

DNA-metabarcoding encompassed most of the diversity observed by CABIN and genera could be aggregated to represent coarser taxonomic reoslution in the other models. Simulating the true distribution of abundance in invertebrate communities carried far greater uncertainty as the proportion of individuals sampled is unknown, and there was no straightforward way to compare occupancy and abundance. Hence the power analysis did not include the N-mixture model based on CABIN abundance data.

The metacommunity

Based on estimates of overall invertebrate genera-diversity (gamma) and occupancy derived from the multi-species occupancy model (MSOM), it is simple to reproduce a presence-absence matrix of the metacommunity from which we can take hypothetical samples. It can however be much more challenging to understand how diversity is distributed in space, and how quickly community composition will change over time. Although flood frequency does relate to geographic location, we regard it, and the other covariates related to temperature, as indicators of temporal change (Figure S2.1). Thus in any given generation of the simulation, occupancy of each taxon in the metacommunity is determined by the environmental conditions proposed (Figure S2.2). Whilst differences in occupancy do result in temporal turnover (approximately 10-27%), this is still not sufficient to explain the rate of turnover observed in the PAD. As a result, we simulated dynamic changes in local community composition (local extinction/colonization) that overlaid the shifts in occupancy due to environmental variation.

Turnover of the presence-absence matrix could be simulated using permutation of occurrences among sites in the simulated metacommunity. This could be continued until the rate of turnover approximated the degree of turnover we observed in the PAD data. For further information about the algorithms available to generate these changes, users should refer to the manual for the function *permat* in the vegan package [31]. Note that although occupancy was allowed to vary between time steps, we preserved the row and column sums during permutation, meaning occupancy and site richness were held constant.

Ultimately, the genus-level turnover in the PAD was so high, on average above 40% (see Figure S1.15), that those observations could only be replicated by the complete redistribution of occurrences. Community assembly at an individual site could therefore be considered purely the result of chance events, with composition of any given site a product of its richness and the relative occupancy of taxa among sites.

Full permutation of presence-absence matrices to generate random assembly patterns is consistent with neutral assembly processes, although given the rate of turnover it is unlikely that dispersal is a limiting factor for the majority of taxa. The near-random dynamics of community assembly could instead be derived from a more complex pattern of neutral processes (random dispersal and stochastic local extinction), niche preferences (supported in part by the significant effects of environmental covariates on the occupancy of some taxa), patch dynamics and mass effects across the PAD landscape [32].

To begin, the first piece of information to structure simulated PAD communities is their overall diversity (i.e. regional gamma diversity): `{r} M`

We then create a matrix to represent 100 possible wetland sites, and their environmental conditions over a number of timesteps, in this case representing years. A hundred sites was large enough to distribute species among sites according to their expected occupancy, but not so large as to be challenging to process. Likewise 20 timesteps was sufficient to test the power to observe any given change without storing and processing excessive volumes of data.

To generate hypothetical environmental conditions for this simulation we used the mean and covariance of the three variables in the original data to simulate new possible values. **Note** that these environmental conditions therefore reflect the data available for the 8 sites in this study, but may not be representative of the variation among all wetlands in the PAD, or of variation outside the six years for which we have data. The three variables that we included in the power analysis were flood frequency, time since ice melt and maximum temperature (see SI Appendix 1 for more details).

```r
library(jagsUI)
library(readxl)
library(knitr)
library(betapart)
library(ecodist)
library(vegan)
library(mvtnorm)
library(mvabund)

# No. of sites
N           = 100
# No. of timesteps
TX          = 20
# Create a blank matrix to record starting occurrence
metacomm    = matrix( 0, nrow=N, ncol=M, dimnames=list(paste0("Site",c(1:N)), pad.lists$Species) )
# Standardize
standardize = function(x){ (x - mean(x))/sd(x)  }
Xcov        = apply(Xcov, 2, standardize)
Xcov        = rmvnorm(mean = apply(Xcov, 2, mean), sig = cov(Xcov), n=TX)
```

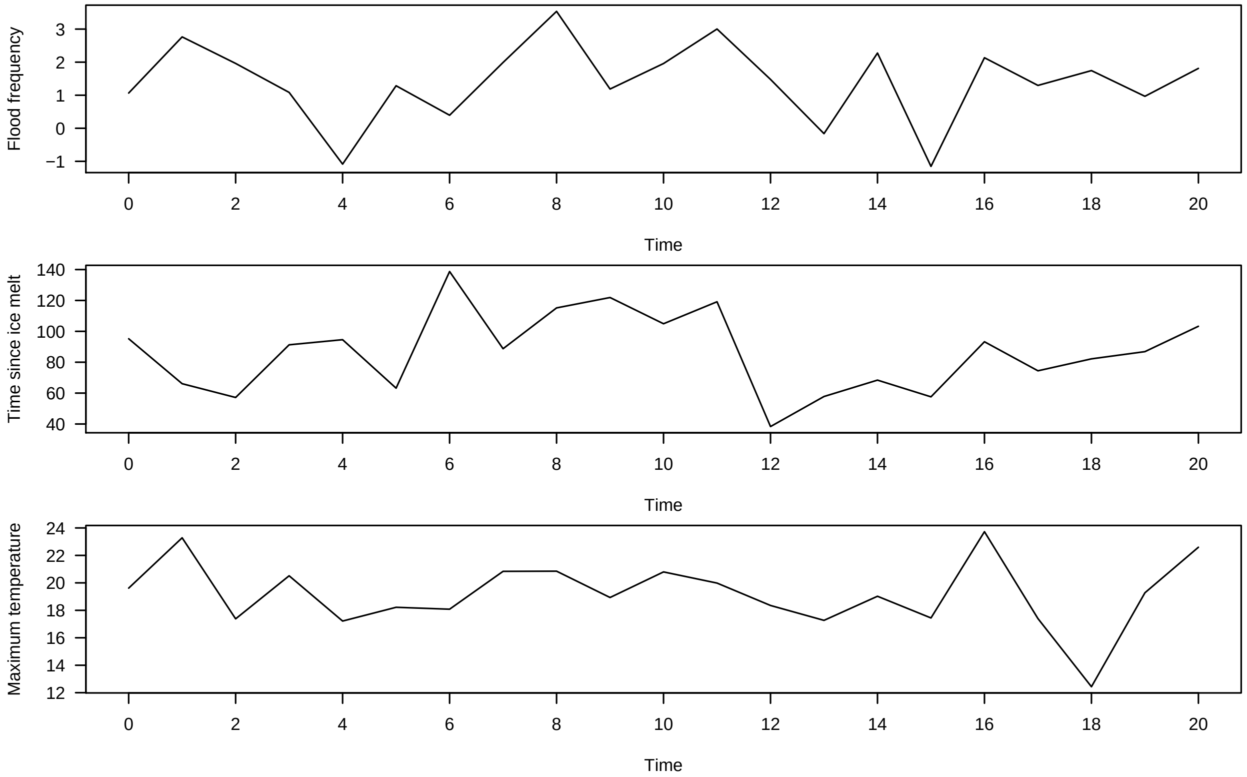

Figure S2.1: Example of a simulated timeseries of the three environmental covariates to determine the expected occupancy of taxa in the genus-level model (see Supplment 1 for more details). Covariates included flood frequency, the time since ice melt, and the maximum temperature prior to survey.

```r
# Create empty table for each species in each time step
spp.occ          = matrix(rep(0,M*TX), nrow=M, ncol=TX)
# Load fitted coefficients
beta             = cbind(MSOM$mean$lpsiS[1:M], MSOM$mean$betalpsiS[1:M,] )
```

```r
# Fill-in expected occupancy (transforming predictions form logistic scale)
for(jj in 1:M){ spp.occ[jj,] = plogis(beta[jj,1] + (beta[jj,2]*Xcov[,1]) +
                              (beta[jj,3]*Xcov[,2]) + (beta[jj,4]*Xcov[,3]) ) }

# Plot
par(mfrow=c(5,5),oma=c(2,2,0.1,0.1),mar=c(2,2,0.5,0.5))
cols= rainbow(M)
xi = rep(seq(1,25,1),M)[1:M]
for(i in 1:25){
  plot(rep(0,TX) ~ c(1:TX), type="l", col="white", ylim=c(0,1),ylab="",las=1,xlab="")
  for(ii in which(xi==i)){ lines(spp.occ[ii,] ~ c(1:TX), col=cols[ii],lwd=2) }
}
mtext("Simulated generations (years)",1,line=0.5,outer=T)
mtext("Predicted occupancy",2,line=0.5,outer=T)
```

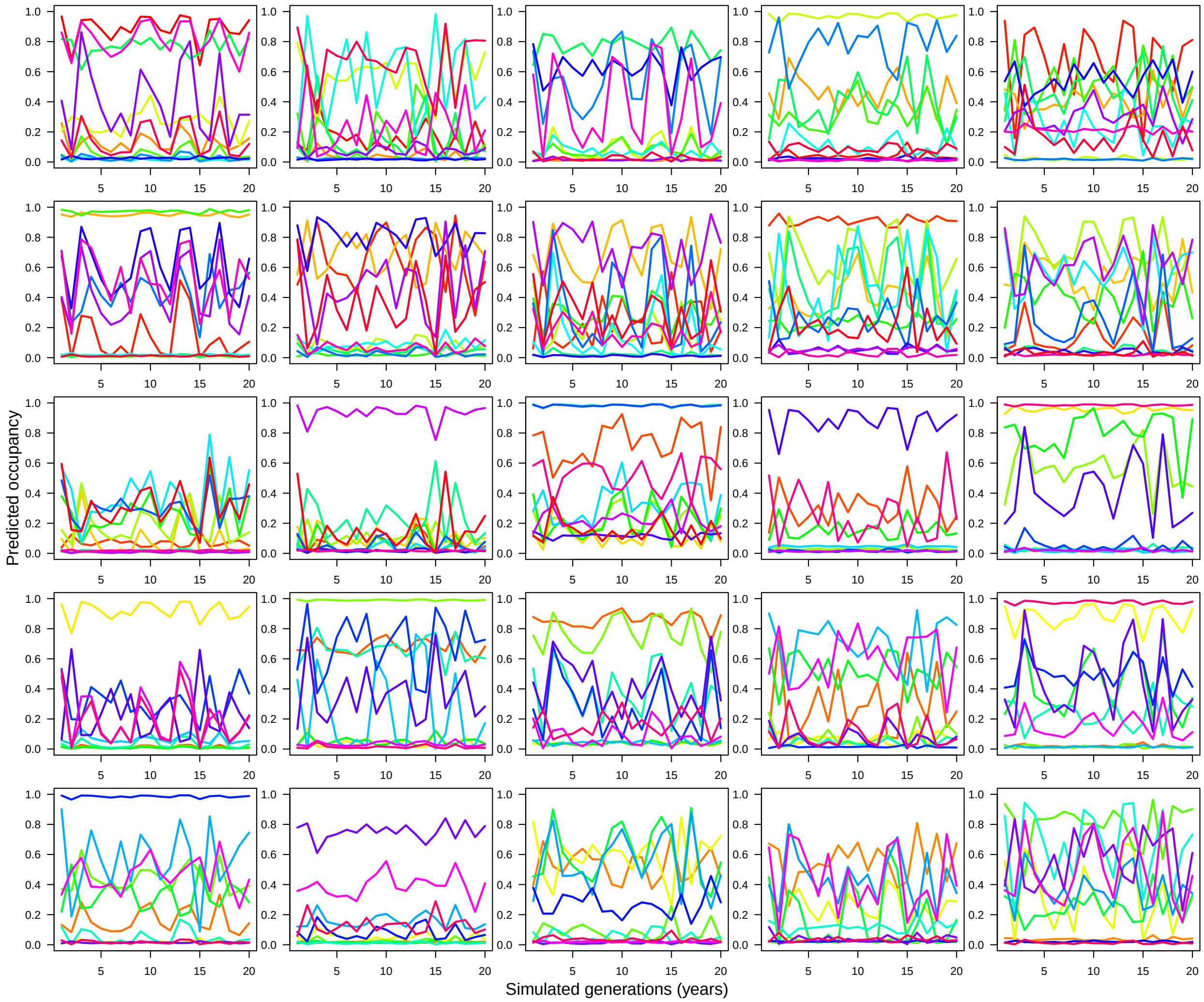

Figure S2.2: Simulated occupancy of 263 genera in response to changes in three environmental covariates over a twenty year period. Parameters were estimated by the hierarchical occupancy model described in SI Appendix 1, based on samples processed with DNA metabarcoding.

```r
# Check all species always occur in at least one site, and if by chance
# they do not, increase so they do so.
```

```
spp.occ[round(spp.occ,2)==0] = 1/N
# Convert proportions to integers of occurrences
spp.occ = round(spp.occ,2) * N

# Create starting values to 'metacomm' for 1st timestep
for(i in 1:M){ metacomm[sample(c(1:N),spp.occ[i,1]),i] = 1 }

# And, working backwards, calculate the proportional change relative to previous generation
occ.delta = spp.occ
for(i in TX:2){ occ.delta[,i] = apply(occ.delta[,c(i-1,i)],1,diff) / (occ.delta[,i-1]/100) }
# There is no change in the first generation
occ.delta[,1] = 0
```

At this stage we reviewed the compositional dissimilarity among samples in space and time expected purely as a result of different probabilities of occurrence and the changes in those probabilities due to environmental responses (Figure S2.3). The turnover among sites in a simulated community (red solid line) falls within the distribution of dissimilarity values observed in the survey data (solid black line). However, the temporal turnover in the simulated community (dashed red line), driven solely by occupancy responses to fluctuating environmental conditions, was typically much lower than the temporal turnover observed in the PAD samples collected (dashed black line). We therefore introduced a process of permutation for the matrix of taxon occurrences which simulates temporal turnover through local extinction and colonisation, but maintains the known distribution of occupancy in the community.

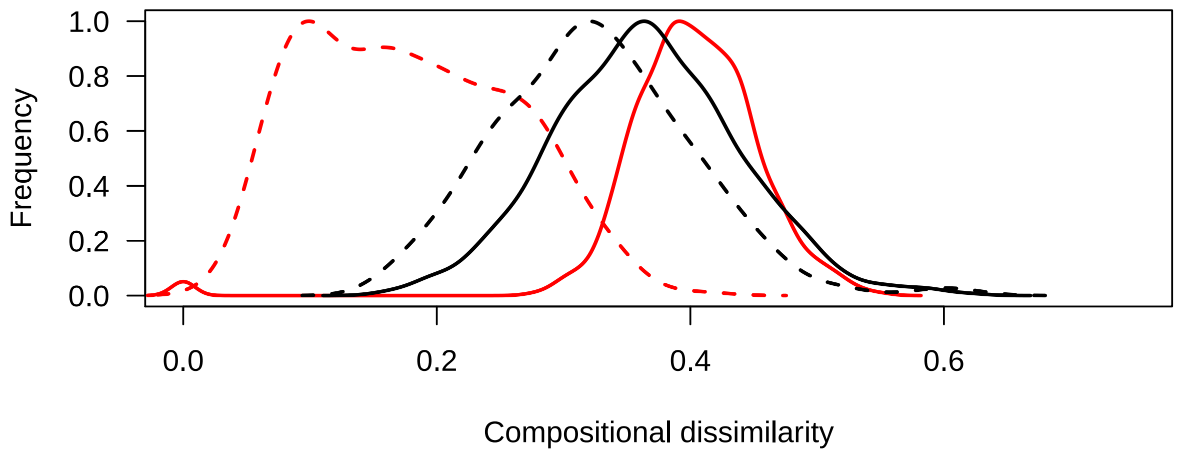

Figure S2.3: Distribution of pairwise dissimilairty among sites in space (solid lines) and over time (dashed lines) for the sites surveyed in the PAD (black lines), and the simulated community composition that replicates the same patterns of occupancy (red lines).

Sensitivity to Impact

As described in the main text, a challenge for estimating our ability to detect a deviation from the expected composition is that we do not know what form that deviation will take. A stressor is unlikely to drive the decline of a community at random. Furthermore, our study was meant to anticipate potential risks, and

therefore we could not easily quantify the magnitude of effects that might be imposed on the ecosystem, such as the distribution and concentration of a given pollutant, or the expected changes in temperature and rainfall patterns. In addition, there is very little information available (e.g. trait data, ecotoxicological trials, field experiments) for how the taxa in this system might respond, particularly for diverse assemblages like invertebrates.

Given we could not use empirical data to assign realistic thresholds, or even approximate the parameters that govern the sensitivity of specific taxa, we modelled the degradation of the metacommunity in response to a hypothetical stressor, hereafter referred to as 'Stressor-X'. Values of stressor-X are thus meaningless. However, what is important is how sensitivity co-varies with taxon's occupancy. One extreme scenario would be for common taxa to also be the most tolerant, and hence identifying the impact of a stressor would rely on observing the loss of taxa with low occupancy (loss of rare taxa). Conversely, the opposite extreme proposed that the most prevalent taxa are the most sensitive to Stressor-X, and therefore the impact of stressor-X is related to the decline of common taxa.

What is missing from the description above, and most other studies, is a consideration of detectability. If species with low tolerance also have a low probability of detection, then changes to the community will be more difficult to confirm with confidence. However, as this study has already modelled the relationship between occupancy and detectability, these characteristics of the community are effectively known. Our statistical power to detect change therefore depends on the magnitude of a shift in composition (the "signal") relative to the background variability of the system (see discussion on fluctuating occupancy and permutation above) and sampling error ("noise" due to imperfect detection).

The simulation function

The simulation function took the following broad steps:
1. To begin, a metacommunity matrix, generated as described above using the parameters estimated by the hierarchical occupancy models, is provided to the function (*start.comm*). As this represents the expected metacommunity at its natural baseline equilibrium, it is referred to as the "null" model.
2. The overall occupancy of each taxon is adjusted in each of *TX* timesteps based on the proportional change described by the *occ.delta* arguement. After this, permutation of the occurrence matrix is used to randomize the assemblage structure and then stored in an array. The final array has *TX* layers equivalent to *start.comm* matrix. This array represents the *null* metacommunity.
3. Repeat the process for a copy of the metacommunity matrix, and then further remove occurrences of taxa whose assigned threshold tolerance (see *SSDx*) falls below the value of Stressor-X (specified by *xgradient*). This array represents the *disturbed* metacommunity.
4. If an argument for *higher.taxa* is provided, the arrays for *null* and *disturbed* occurrence are aggregated to reflect occurrence of their parent taxonomic rankings; in this case genus to family-level.
5. New matrices are then created to represent the **observed** composition of both the *null* and *disturbed* metacommunities given a) the detectability of the taxa, and b) the number of samples taken.
6. Finally the compositional dissimilarity of the surveyed sites in the *null* and *disturbed* arrays is compared in each annual time step using *mvabund*. For the purposes of this study we were interested in how many samples would be required to detect a particular (p<0.05) shift in composition with a given degree of confidence. The number of samples required to detect shifts in community composition in any given year was very high. As a result, for the purposes of this study we studied the power required to detect a significant shift at least 50% of the time (i.e. on average within 2 years). Significant differences therefore needed to be observed in half the TX steps to be considered successful. As this testing phase was the most time consuming step, a further argument (*mva.stop*) was added to the routine so that the function would escape if this desired threshold was met (i.e. the no. of significant results was equivalent to TX/2).

```
metacomm.permute = function(TX,                  # Total no. of time steps (years)
                            start.comm,          # Starting compositional matrix
                            occ.delta,           # Proportional change (+/-) in occupancy.
```

```
                             higher.taxa = NULL, # Table of taxon names and higher ranks.
                             # Disturbance parameters
                             SSDx        = NULL,   # Species Sensitivity Distribution
                             xgradient = NULL,   # Vector of 'StressorX'.
                             # Observation parameters
                             survey,              # Index of surveyed sites (rows)
                             detection = NULL,   # List of detectabilities and reps
                             mva.stop   = NULL
){
  # No. of taxa and sites
  M = ncol(start.comm)
  N = nrow(start.comm)

  # - - - - - - - - - - - - - - - - - - - - - - - - - - - #
  # CREATE NULL METACOMMUNITY
  # - - - - - - - - - - - - - - - - - - - - - - - - - - - #

  # Create null array to record composition (site X species) in TX annual time steps
  tcomp = array(0, dim=c(N,M,TX), dimnames=list(paste0("Site",c(1:N)),
                                                 paste0("Spp", c(1:M)),
                                                 paste0("Time",c(1:TX))) )
  # Composition at time 1 is equivalent to 'start.comm'
  tcomp[,,1] = start.comm

  # Loop over time steps
  for(i in 2:TX){
    # Start with composition of previous time-step
    comp = tcomp[,,i-1]
    # Account for any changes in species' occupancy
    for(ii in which(occ.delta[,i]!=0) ){
      # Note: proportional changes must be rounded to nearest whole number
      xi = round((sum(comp[,ii])/100) * (occ.delta[ii,i]))
      if( xi>0 ){
        xii = which(comp[,ii]==0)
        xi  = min(xi,length(xii))
        comp[sample(xii,abs(xi)),ii] = 1
      }
      if( xi<0 ){
        xii = which(comp[,ii]==1)
        xi  = min(abs(xi),length(xii))
        comp[sample(xii,abs(xi)),ii] = 0
      }
    }
    # Permutation
    X = permatswap(comp,method="tswap",fixedmar="both",mtype="prab",
                   times=1,burnin=150000,thin=1000)
    # Enter matrix with new composition into the next timestep of array
    tcomp[,,i] = X$perm[[1]]
  } # End TX loop

  # - - - - - - - - - - - - - - - - - - - - - - - - - - - #
  # OVERLAY DISTURBANCE ON EQUIVALENT METACOMMUNITY
  # - - - - - - - - - - - - - - - - - - - - - - - - - - - #
```

```
# Create an equivalent metacommunity array to record responses to Stressor-X.
dcomp     = tcomp
dcomp[,,] = 0
# If stress isn't specified for each year, use the first value for all years
if(length(xgradient)!=TX){
  xgradient = rep(xgradient[1],TX)
  print("Using first value of xgradient for all TX years.")
}

# Loop over time steps
for(i in 1:TX){
  # Start with composition of previous time-step
  # Note unlike the null routine above is that we now loop over step 1 too
  # and hence must make sure here that we don't refer to a previous timestep.
  if(i>1){ comp = dcomp[,,i-1] } else { comp = start.comm }

  # Account for any changes in species' occupancy
  for(ii in which(occ.delta[,i]!=0) ){
    # Note: proportional changes must be rounded to nearest whole number
    xi = round((sum(comp[,ii])/100) * (occ.delta[ii,i]))
    if( xi>0 ){
      xii = which(comp[,ii]==0)
      xi  = min(xi,length(xii))
      comp[sample(xii,abs(xi)),ii] = 1
    }
    if( xi<0 ){
      xii = which(comp[,ii]==1)
      xi  = min(abs(xi),length(xii))
      comp[sample(xii,abs(xi)),ii] = 0
    }
  }

  # Remove occurrences from community below a certain threshold
  # Which taxa are below the threshold for year 'i'?
  xid = which(SSDx < xgradient[i])
  # Set occurrences to zero
  comp[,xid] = 0
  # Permutation
  X = permatswap(comp,method="tswap",fixedmar="both",mtype="prab",
                 times=1,burnin=150000,thin=1000)
  # Store output
  dcomp[,,i] = X$perm[[length(X$perm)]]

} # End TX loop


# - - - - - - - - - - - - - - - - - - - - - - - - - - - - #
# AGGREGATE THE GENUS-LEVEL COMMUNITY TO FAMILY-LEVEL TAXA
# - - - - - - - - - - - - - - - - - - - - - - - - - - - - #

if( !is.null(higher.taxa) ){
  # 'HR' is for 'higher rank'. Create arrays the right size
  HRtcomp     = tcomp[,1:length(unique(higher.taxa[,1])),]
```

```
  HRdcomp     = dcomp[,1:length(unique(higher.taxa[,1])),]

  # Now aggregate occurrences to higher taxon rank groups.
  for(i in 1:TX){
    HRtcomp[,,i] = t(aggregate(t(tcomp[,,i]), by=list(higher.taxa[,1]), FUN=max)[,-1])
    HRdcomp[,,i] = t(aggregate(t(dcomp[,,i]), by=list(higher.taxa[,1]), FUN=max)[,-1])
  }
  tcomp = HRtcomp ; rm(HRtcomp)
  dcomp = HRdcomp ; rm(HRdcomp)
}

# - - - - - - - - - - - - - - - - - - - - - - - - - - - - #
# APPLY DETECTION ERROR TO EACH METACOMMUNITY
# - - - - - - - - - - - - - - - - - - - - - - - - - - - - #

# Arguement 'detection' includes detectability with each mode of observation and the
# number of replicate samples using that method.
det1  = detection[[1]]
reps1 = detection[[2]]
det2  = detection[[3]]
reps2 = detection[[4]]

# Overlap detectabilities on occurrence matrix based on different levels of
# sampling ('reps') and processing effort (separate primers)
tcomp.obs = tcomp
dcomp.obs = dcomp
for(i in 1:TX){
  if( reps1>0 ){
    simcomm.t1 = rbinom(tcomp[,,i], reps1, det1) * tcomp[,,i]
    simcomm.d1 = rbinom(dcomp[,,i], reps1, det1) * dcomp[,,i]
  } else {
    simcomm.t1 = simcomm.d1 = matrix(0,N,M)
  }
  if( reps2>0 ){
    simcomm.t2 = rbinom(tcomp[,,i], reps2, det2) * tcomp[,,i]
    simcomm.d2 = rbinom(dcomp[,,i], reps2, det2) * dcomp[,,i]
  } else {
    simcomm.t2 = simcomm.d2 = matrix(0,N,M)
  }
  # Combine
  simcomm.t = simcomm.t1 + simcomm.t2 ; simcomm.t[simcomm.t>0] = 1
  simcomm.d = simcomm.d1 + simcomm.d2 ; simcomm.d[simcomm.d>0] = 1
  # Fill array
  tcomp.obs[,,i] = simcomm.t
  dcomp.obs[,,i] = simcomm.d

  rm(simcomm.t1,simcomm.t2,simcomm.d1,simcomm.d2,simcomm.t,simcomm.d)
}

# - - - - - - - - - - - - - - - - - - - - - - - - - - - - - - #
# SUMMARY STATS
# - - - - - - - - - - - - - - - - - - - - - - - - - - - - - - #
```

```
  # Test for differences between null and degraded communities with mvabund

  # Can compare groups of sites from A SINGLE year:
  mv.anova = data.frame("TX"=1:TX,"Dev.null"=NA,"p.null"=NA,"Beta.null"=NA,
                        "Dev.obs"=NA,"p.obs"=NA,"Beta.obs"=NA)
  if(is.null(mva.stop)){ mva.stop = TX }

  for(i in 1:TX){
    # The 'mva.stop' argument specifies an escape so that the function stops calculating significance a
    if( sum(mv.anova$p.obs[!is.na(mv.anova$p.obs)]<=0.05)<mva.stop ){
      inv.spp = mvabund(rbind(tcomp[survey,,i],dcomp[survey,,i])) # Format data for 'mvabund'
      xgroup  = factor(rep(c("A","B"),each=length(survey)))       # Create 'treatement' factor
      xi      = manyglm( inv.spp ~ xgroup, family="binomial")
      xii     = anova(xi)
      rowindex= c(1:length(survey))+length(survey)
      nrow    = length(survey)*2
      colindex= c(1:length(survey))
      Beta    = mean( as.matrix(vegdist(inv.spp))[rowindex + nrow * (colindex-1)])
      mv.anova[i,c("Dev.null","p.null","Beta.null")] = c(xii$table$Dev[2],xii$table$`Pr(>Dev)`[2],Beta)

      # Repeat for observed matrices
      inv.spp = mvabund(rbind(tcomp.obs[survey,,i],dcomp.obs[survey,,i]))
      xi = manyglm( inv.spp ~ xgroup, family="binomial")
      xii = anova(xi)
      Beta = mean( as.matrix(vegdist(inv.spp))[rowindex + nrow * (colindex-1)])
      mv.anova[i,c("Dev.obs","p.obs","Beta.obs")] = c(xii$table$Dev[2],xii$table$`Pr(>Dev)`[2],Beta)
    }
  }

  # - - - - - - - - - - - - - - - - - - - - - - - - - - - - - #
  # SAVE
  # - - - - - - - - - - - - - - - - - - - - - - - - - - - - - #

  # Save as list
  tdat = list("TX"          = TX,
              "N"           = N,
              "M"           = M,
              "Stress"      = list("SSDx"      = SSDx,
                                   "xgradient" = xgradient),
              "Survey"      = survey,
              "MVA"         = mv.anova,
              "Permutation" = list(tcomp,tcomp.obs,dcomp,dcomp.obs) )

  # - - - - - - - - - - - - - - - - - - - - - - - - - - - - - #
  # - - - - - - - - - - - - - - - - - - - - - - - - - - - - - #

  return(tdat)
}
```

For the purposes of this example we wished to illustrate a case where the degree of degradation in the metacommunity was not consistently detected in every single year based on the same sample size (8 sites) as was available in our dataset. We therefore ran the code below applying a stressor threshold that reduced the

overall number of occurrences in the metacommunity by 18% (see *Stress.prop*=0.18), and specify a neutral relationship (*SSD.cor*=0) between occupancy and tolerance to Stressor-X.

```
# - - - - - - - - - - - - - - - - #
# SAMPLE SIZE
# - - - - - - - - - - - - - - - - #

# As example, sample 8 of the N sites (rows) in metacomm
Survey.sites = sample(c(1:N),8)

# - - - - - - - - - - - - - - - - #
# GET PREDICTED DETECTABILITY
# - - - - - - - - - - - - - - - - #

# Draw estimates of detectability from the occupancy model
det1      = matrix(rep(plogis(MSOM$mean$lpS[1:M,1]),e=N),nrow=N) # BR5
det2      = matrix(rep(plogis(MSOM$mean$lpS[1:M,2]),e=N),nrow=N) # F230

# Note that a detection argument of 'list(det1,1,det2,1)' indicates the community was sampled with each
# For comparison with morphological approaches we simply specify no samples in the last entry: 'list(de

# - - - - - - - - - - - - - - - - #
# SSD
# - - - - - - - - - - - - - - - - #

SSD.cor = 0

standard01.complement = function(y, rho, x) {
  range01 = function(x){ (x-min(x))/(max(x)-min(x)) }
  if(missing(x)){ x = rnorm(length(y)) } # Optional: supply a default if `x` is not given
  yp   = residuals(lm(x ~ y))
  q    = rho * sd(yp) * y + yp * sd(y) * sqrt(1 - rho^2)
  q    = range01(q)
  return(q)
}

SSDx = standard01.complement(y=spp.occ[,1], rho=SSD.cor, x=runif(M,0,1)) # SSDx vector with desired cor



# Identify the value of StressorX that affects the target proportion of the occurrences for the communi
# As the frqeuency of different tolerances varies the quantile is weighted by occupancy.
Stress.prop = 0.18
StressX     = unlist(lapply(SSDx, FUN=function(x) rep(x,e=as.numeric(colSums(metacomm))[match(x,SSDx)])
StressX     = quantile(StressX, Stress.prop)

# Run simulation

MP = metacomm.permute(TX,
                      start.comm    = metacomm,
                      occ.delta,
                      higher.taxa   = NULL,
                      SSDx,
                      xgradient     = rep(StressX,TX),
```

```
                          survey          = Survey.sites,
                          detection       = list(det1,1,det2,1),
                          mva.stop        = NULL)
```

```
## Time elapsed: 0 hr 0 min 20 sec
## Time elapsed: 0 hr 0 min 20 sec
## Time elapsed: 0 hr 0 min 19 sec
## Time elapsed: 0 hr 0 min 20 sec
## Time elapsed: 0 hr 0 min 19 sec
## Time elapsed: 0 hr 0 min 19 sec
## Time elapsed: 0 hr 0 min 18 sec
## Time elapsed: 0 hr 0 min 19 sec
## Time elapsed: 0 hr 0 min 18 sec
## Time elapsed: 0 hr 0 min 19 sec
## Time elapsed: 0 hr 0 min 18 sec
## Time elapsed: 0 hr 0 min 20 sec
## Time elapsed: 0 hr 0 min 18 sec
## Time elapsed: 0 hr 0 min 19 sec
## Time elapsed: 0 hr 0 min 19 sec
## Time elapsed: 0 hr 0 min 19 sec
## Time elapsed: 0 hr 0 min 18 sec
## Time elapsed: 0 hr 0 min 19 sec
## Time elapsed: 0 hr 0 min 18 sec
## Time elapsed: 0 hr 0 min 19 sec
## Time elapsed: 0 hr 0 min 19 sec
## Time elapsed: 0 hr 0 min 19 sec
## Time elapsed: 0 hr 0 min 19 sec
## Time elapsed: 0 hr 0 min 20 sec
## Time elapsed: 0 hr 0 min 18 sec
## Time elapsed: 0 hr 0 min 19 sec
## Time elapsed: 0 hr 0 min 18 sec
## Time elapsed: 0 hr 0 min 19 sec
## Time elapsed: 0 hr 0 min 19 sec
## Time elapsed: 0 hr 0 min 19 sec
## Time elapsed: 0 hr 0 min 19 sec
## Time elapsed: 0 hr 0 min 19 sec
## Time elapsed: 0 hr 0 min 20 sec
## Time elapsed: 0 hr 0 min 20 sec
## Time elapsed: 0 hr 0 min 19 sec
## Time elapsed: 0 hr 0 min 20 sec
## Time elapsed: 0 hr 0 min 20 sec
## Time elapsed: 0 hr 0 min 21 sec
## Time elapsed: 0 hr 0 min 19 sec
## Time elapsed: 0 hr 0 min 20 sec
```

Simulation results

To begin looking at how the outputs of the simulation were interpreted, Figure S2.4 summarises how the overall number of occurrences in the sampled sites were expected to vary naturally over a 20 year period. As this is the true state expected by the baseline surveys, we refered to it as the *Null process* model. The *Null observed* model shows the number of occurrences we would then expect to detect given the detectability of the

taxa. Based on the simulated introduction of stressor-X, and an approximate 18% reduction in occurrences (see above), the red lines display the equivalent relative number of occurrences for the *Stressed process* model and *Stressed observed* model.

The red dots in Figure S2.4 indicate when there is a significant difference between the process data models, or observed data models, as identified by the multivariate generalized linear models (see *mvabund* package). As shown, with perfect detection and recovery of all taxa present, the differences in composition between the *null-process* and *stressed-process* models were consistently detected. Once we account for detectability the differences between the communities were captured more intermittently. As mentioned, this power analysis was used to identify at what value of *Stress.prop* were the differences in the observed data likely to be detected at least 50% of the time.

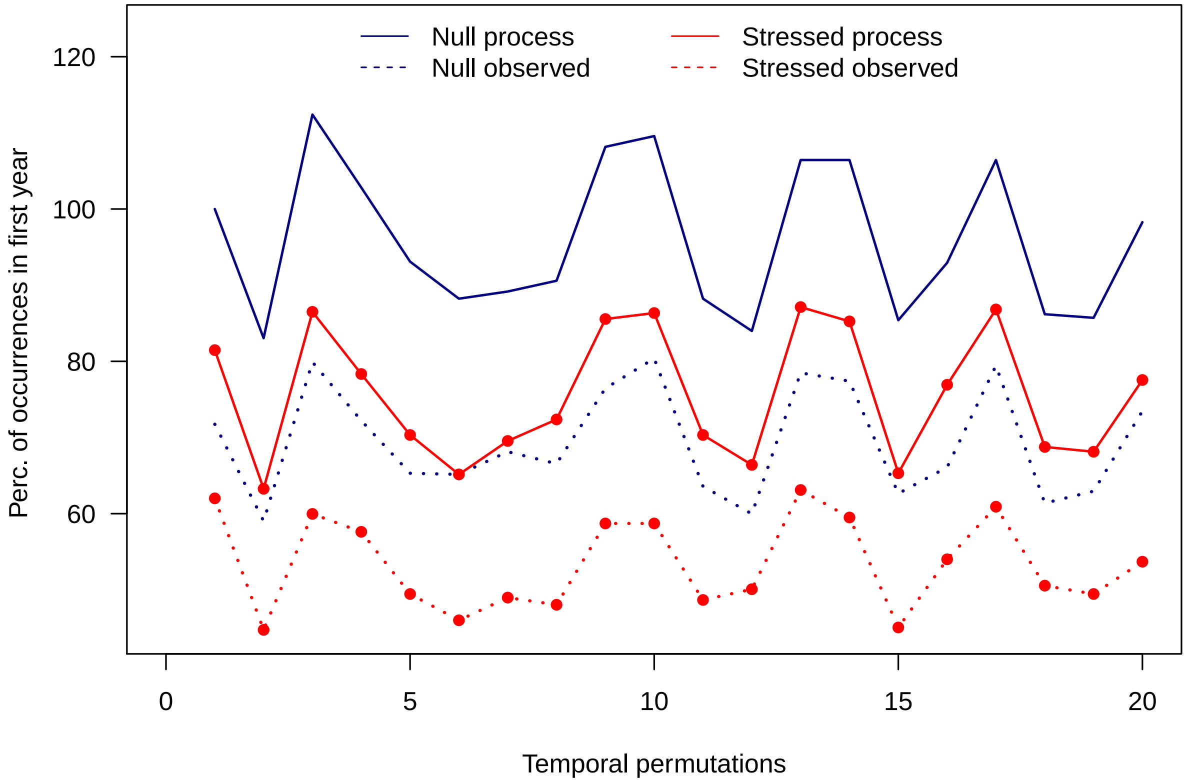

Figure S2.4: Proportion of occurrences sampled relative to the number present at the start of the simulated null community. The blue solid line represents the null model of the baseline metacommunity processes, and the red solid line depicts the same community impacted by a selected degree of stress, which in this case reduces the number of occurrences by 18%. The dotted lines represent the fraction of occurrences observed in samples from null and stressed communities. A red dot marks when the composition of the process or observed models were significantly different from each other.

Another option would be to compare how dissimilar the composition of *null* and *degraded* communities would be, and how dissimilar they appear to be after accounting for detectability.

```
XYLIM = range(MP$MVA$Beta.obs,MP$MVA$Beta.null)
plot(MP$MVA$Beta.obs ~ MP$MVA$Beta.null,xlim=XYLIM,ylim=XYLIM,
```

```
     xlab = "True dissimilarity of sampled sites",
     ylab = "Observed dissimilarity of sampled sites", las=1)
abline(0,1)
xi = which(MP$MVA$p.null<=0.05)
for(i in xi){ lines(x=rep(MP$MVA$Beta.null[i],2), y=c(MP$MVA$Beta.obs[i]-0.001,MP$MVA$Beta.obs[i]-0.005[illegible]
xi = which(MP$MVA$p.obs<=0.05)
for(i in xi){ lines(x=c(MP$MVA$Beta.null[i]-0.001,MP$MVA$Beta.null[i]-0.005),
                    y=rep(MP$MVA$Beta.obs[i],2),lwd=2,col="red" ) }
points(MP$MVA$Beta.obs ~ MP$MVA$Beta.null, pch=16)
```

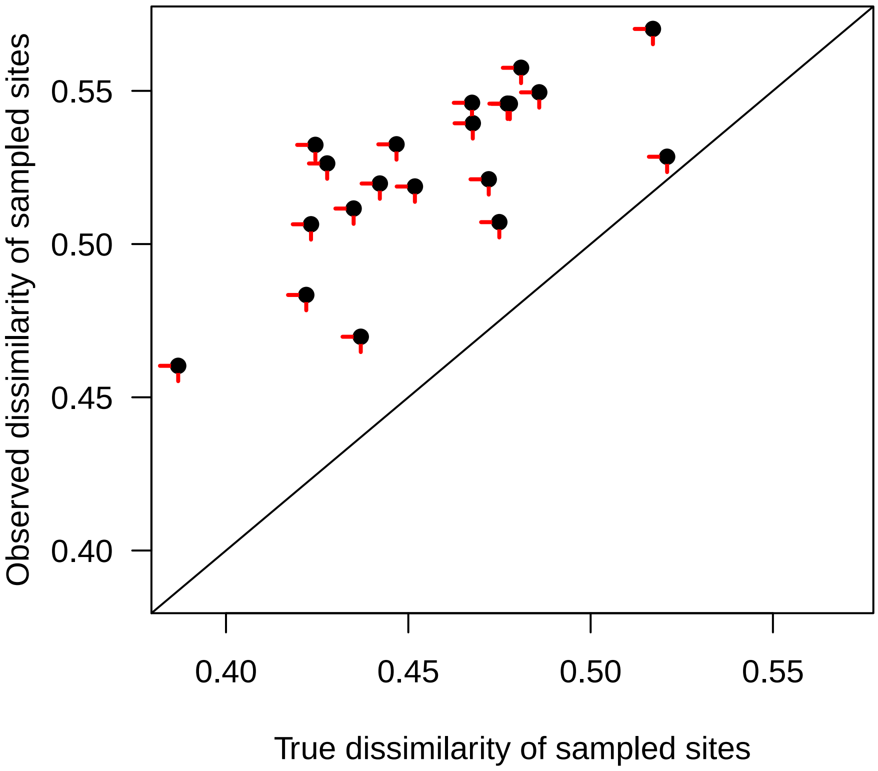

Figure S2.5: Relationship between the true and observed compositional dissimilarity between sampled sites for each simulated year. If composition was significantly different between the expected null community and the stressed equivalent a vertical red tick is added to the point (see points on solid red line in Figure S2.4). Likewise, significant differences between the metacommunities after accounting for detectability are indicated with a horizontal red tick (see points on dotted red line in Figure S2.4).

Given the observed spatial and temporal turnover in composition, it would be unrealistic to expect surveys to reliably detect differences from the expected null community in **any** year. Therefore, sampling was considered adequate if the stressed community was detectably different from the expected null community at least 50% of the time. We subsequently repeated the simulation 100 times for each combination of sample size and correlation with occupancy to account for variation in the assigned occupancy, tolerance thresholds and environmental parameters. If too many simulations failed to meet the desired threshold, the magnitude of Stressor-X was increased, further reducing the number of occurrences in the stressed community. The

reduction in taxon occupancy was stopped once significant differences could be identified in 50% of years with 95% confidence, (i.e. in >95 simulations).

Below, Figure S2.6 describes how the threshold for detection was moderated by both sample size and the correlation with detectability. For example, a reduction in occurrences of approximately 10% could be detected by 7 samples per year **IF** a taxon's tolerance to a particular factor ('Stressor-X') was perfectly negatively correlated with its occupancy (i.e. common taxa are least tolerant, and hence first to be affected by increased stress). Conversely if rare taxa are most sensitive to stress (positive correlation in Figure S2.6 x-axis), then 12-14 samples may be required to detect the same 10% reduction in occupancy. Note that these sample sizes are not generic recommendations; they are specific to the PAD metacommunity, because the distribution of occupancy and correlation of occupancy with detectability will influence statistical power.

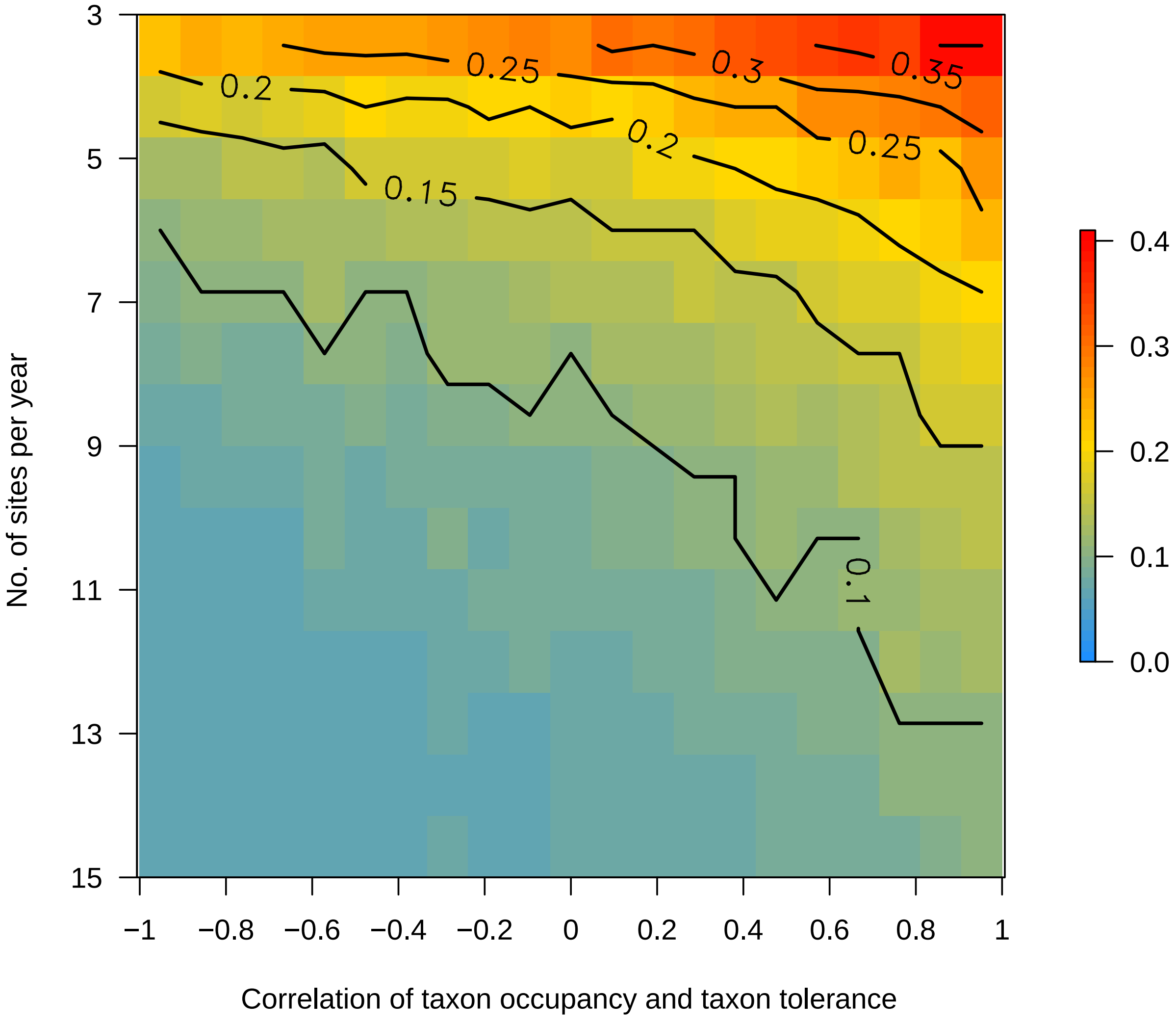

Figure S2.6: Minimum reduction to community occupancy detectable in 50% of years, with 95% confidence. Plot shows relationship between the number of sites sampled per year (a single sample per site), and the correlation of community tolerance with occupancy (see *Sensitivity to Impact* section above), upon the magnitude of change to the PAD metacommunity that can be detected.

Finally we compared the power to detect a reduction in occupancy, relative to the 'null' baseline metacommunity model, using different approaches to data generation. Figure S2.7 demonstrates that our power to confidently detect changes in occupancy increases both as sample size increases (either by increasing the number of sites per year or increasing within-site replication), and as the data resolution increases, specifically improving from CABIN family data to DNA-based family data, and again to the DNA data at genus-level.

For example, even surveying 15-20 sites per year (a higher level of sampling effort than has been possible to date), we would not expect CABIN data to reliably detect reductions in occupancy less than about 20%, whereas DNA metabarcoding at the genus-level could detect reductions close to 5%.

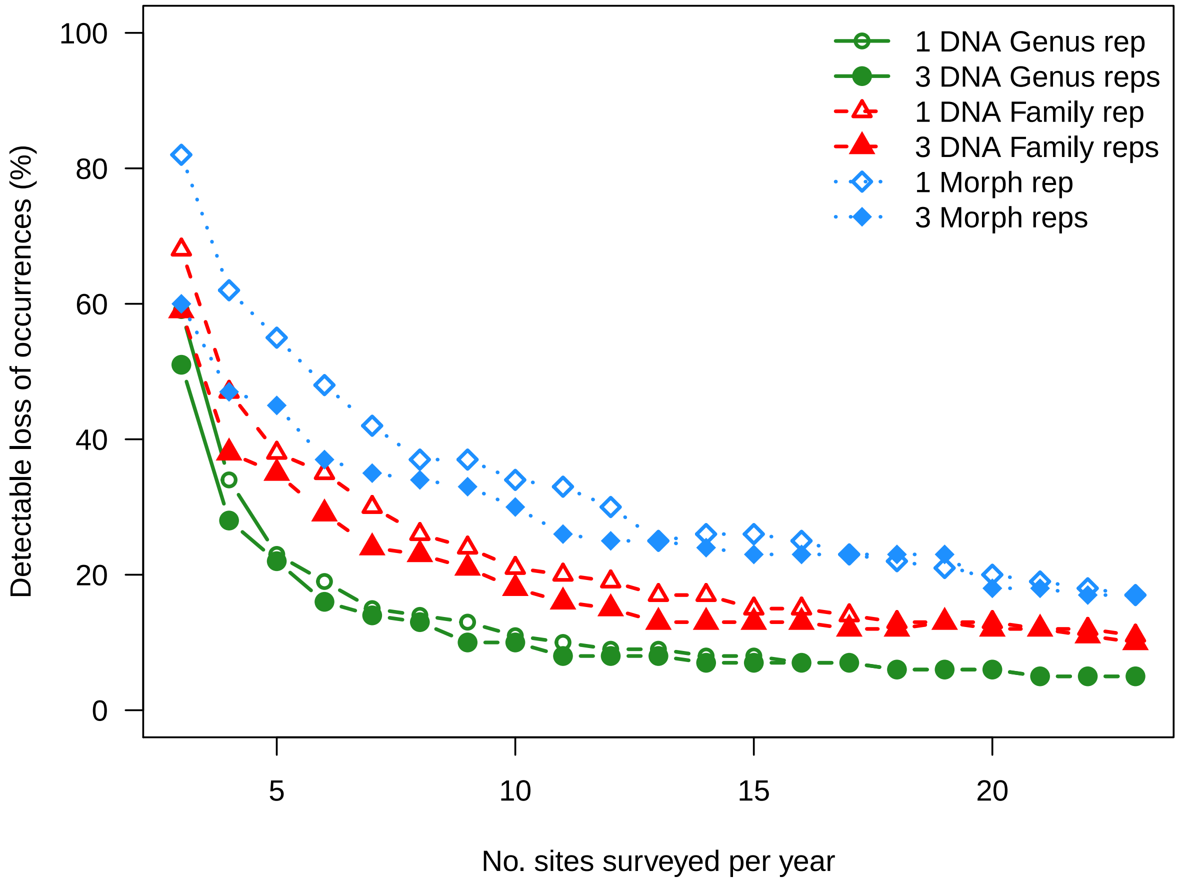


```
## pdf
##   2
```

Figure S2.7: Minimum detectable reduction of community occupancy (in 50% of years with 95% confidence) in response to number of annual survey sites. The threshold at which changes could be detected was influenced by approach to processing samples, the taxonomic resolution, and the number of replicate samples at each site surveyed.

Supporting Information References

1. Hajibabaei M, Spall JL, Shokralla S, & van Konynenburg S (2012) Assessing biodiversity of a freshwater benthic macroinvertebrate community through non-destructive environmental barcoding of DNA from preservative ethanol. *BMC Ecology* 12:28-28.
2. Gibson J*, et al.* (2014) Simultaneous assessment of the macrobiome and microbiome in a bulk sample of tropical arthropods through DNA metasystematics. *Proceedings of the National Academy of Sciences* 111(22):8007-8012.
3. Folmer O, Black M, Hoeh W, Lutz R, & Vrijenhoek R (1994) DNA primers for amplification of mitochondrial cytochrome c oxidase subunit I from diverse metazoan invertebrates. *Mol. Marine Biol. Biotech.* 3:294-299.
4. Gibson JF*, et al.* (2015) Large-Scale Biomonitoring of Remote and Threatened Ecosystems via High-Throughput Sequencing. *PLoS ONE* 10(10):e0138432.
5. Peters DL, Prowse TD, Pietroniro A, & Leconte R (2006) Flood hydrology of the Peace-Athabasca Delta, northern Canada. *Hydrological Processes* 20(19):4073-4096.
6. Peters DL, Caissie D, Monk WA, Rood SB, & St-Hilaire A (2016) An ecological perspective on floods in Canada. *Canadian Water Resources Journal / Revue canadienne des ressources hydriques* 41(1-2):288-306.
7. Tange O (2011) GNU Parallel: The Command-Line Power Tool. *;login* 36(1):42-47.
8. St.John J (2016) SeqPrep).
9. Martin M (2011) Cutadapt removes adapter sequences from high-throughput sequencing reads. *EMBnet.journal* 17(1):10-12.
10. Rognes T, Flouri T, Nichols B, Quince C, & Mahé F (2016) VSEARCH: a versatile open source tool for metagenomics. *PeerJ* 4:e2584.
11. Edgar RC (2016) UNOISE2: improved error-correction for Illumina 16S and ITS amplicon sequencing. *bioRxiv*.
12. Porter TM & Hajibabaei M (2018) Automated high throughput animal CO1 metabarcode classification. *Scientific Reports* 8(1):4226.
13. Wang Q, Garrity GM, Tiedje JM, & Cole JR (2007) Naive Bayesian classifier for rapid assignment of rRNA sequences into the new bacterial taxonomy. *Applied and environmental microbiology* 73(16):5261-5267.
14. Guillera-Arroita G (2017) Modelling of species distributions, range dynamics and communities under imperfect detection: advances, challenges and opportunities. *Ecography* 40(2):281-295.
15. Kery M & Royle AJ (2009) Inference about species richness and community structure using species-specific occupancy models in the national Swiss breeding bird survey MHB. *Modeling demographic processes in marked populations. Environmental and ecological statistics, Vol. 3.*, eds Thomson DL, Cooch EG, & Conroy MJ (Springer, New York).
16. Kéry M & Royle AJ (2015) *Applied Hierarchical Modeling in Ecology: Analysis of distribution, abundance and species richness in R and BUGS: Volume 1: Prelude and Static Models* (Academic Print).
17. Cao Y, Hawkins CP, & Vinson MR (2003) Measuring and controlling data quality in biological assemblage surveys with special reference to stream benthic macroinvertebrates. *Freshwater Biology* 48(10):1898-1911.
18. Singer GAC, Fahner NA, Barnes JG, McCarthy A, & Hajibabaei M (2019) Comprehensive biodiversity analysis via ultra-deep patterned flow cell technology: a case study of eDNA metabarcoding seawater. *Scientific Reports* 9(1):5991.

19. Dorazio RM, Royle JA, Söderström B, & Glimskär A (2006) Estimating species richness and accummulation by modelling species occurrence and detectability. *Ecology* 87(4):842–854.
20. Guillera-Arroita G, Kéry M, & Lahoz-Monfort JJ (2019) Inferring species richness using multispecies occupancy modeling: Estimation performance and interpretation. *Ecology and Evolution* 9(2):780-792.
21. Gelman A & Rubin DB (1992) Inference from Iterative Simulation using Multiple Sequences. *Statistical Science* 7:457-511.
22. Warton DI, Stoklosa J, Guillera-Arroita G, MacKenzie DI, & Welsh AH (2017) Graphical diagnostics for occupancy models with imperfect detection. *Methods in Ecology and Evolution* 8(4):408-419.
23. MacKenzie DI & Bailey LL (2004) Assessing the fit of site-occupancy models. *Journal of Agricultural, Biological, and Environmental Statistics* 9(3):300-318.
24. Royle JA, Kéry M, Gautier R, & Schmid H (2007) Hierarchical spatial models of abundance and occurrence from imperfect survey data. *Ecological Monographs* 77(3):465-481.
25. Kuo L & Mallick B (1998) Variable selection for regression models. *Sankhya: The Indian Journal of Statistics, Series B (1960–2002)* 60(65-81).
26. Bush A*, et al.* (2019) Studying Ecosystems With DNA Metabarcoding: Lessons From Biomonitoring of Aquatic Macroinvertebrates. *Frontiers in Ecology and Evolution* 7(434).
27. Yamaura Y*, et al.* (2011) Modelling community dynamics based on species-level abundance models from detection/nondetection data. *Journal of Applied Ecology* 48(1):67-75.
28. Elbrecht V, Peinert B, & Leese F (2017) Sorting things out: Assessing effects of unequal specimen biomass on DNA metabarcoding. *Ecology and Evolution* 7(17):6918-6926.
29. Curry CJ, Gibson JF, Shokralla S, Hajibabaei M, & Baird DJ (2018) Identifying North American freshwater invertebrates using DNA barcodes: are existing COI sequence libraries fit for purpose? *Freshwater Science* 37(1):178-189.
30. Bonada N, Prat N, Resh VH, & Statzner B (2006) Developments in Aquatic Insect Biomonitoring: A Comparative Analysis of Recent Approaches. *Annual Review of Entomology* 51:495–523.
31. Oksanen J*, et al.* (2019) vegan: Community Ecology Package. R package version 2.5-4.).
32. Leibold M & Chase J (2018) *Metacommunity Ecology* (Princeton University Press, Princeton and Oxford).
